# The Autoimmune Ecology

**DOI:** 10.3389/fimmu.2016.00139

**Published:** 2016-04-26

**Authors:** Juan-Manuel Anaya, Carolina Ramirez-Santana, Maria A. Alzate, Nicolas Molano-Gonzalez, Adriana Rojas-Villarraga

**Affiliations:** ^1^Center for Autoimmune Diseases Research (CREA), School of Medicine and Health Sciences, Universidad del Rosario, Bogotá, Colombia

**Keywords:** environment, ecology, autoimmune disease, polyautoimmunity, personalized medicine

## Abstract

Autoimmune diseases (ADs) represent a heterogeneous group of disorders that affect specific target organs or multiple organ systems. These conditions share common immunopathogenic mechanisms (i.e., the autoimmune tautology), which explain the clinical similarities they have among them as well as their familial clustering (i.e., coaggregation). As part of the autoimmune tautology, the influence of environmental exposure on the risk of developing ADs is paramount (i.e., the autoimmune ecology). In fact, environment, more than genetics, shapes immune system. Autoimmune ecology is akin to exposome, that is all the exposures – internal and external – across the lifespan, interacting with hereditary factors (both genetics and epigenetics) to favor or protect against autoimmunity and its outcomes. Herein, we provide an overview of the autoimmune ecology, focusing on the immune response to environmental agents in general, and microbiota, cigarette smoking, alcohol and coffee consumption, socioeconomic status (SES), gender and sex hormones, vitamin D, organic solvents, and vaccines in particular. Inclusion of the autoimmune ecology in disease etiology and health will improve the way personalized medicine is currently conceived and applied.

## Introduction

Public health problems are increasingly complex, especially environmental and social threats arising from global changes, driven by rapid industrialization, population growth, over-consumption of natural resources, and inappropriate use of technology (1). Understanding patterns of health and disease requires that public health attention be given to not only personal behaviors, biological traits, and specific risks but also the social and physical environmental characteristics that shape human experience (1).

**Figure 1:**
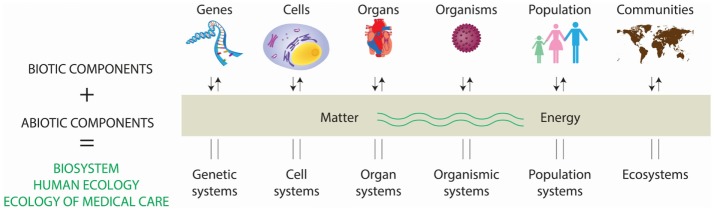
**Interaction of living and non-living components in the conformation of the biosystem**. A system consist of regularly and interdependent components forming a unified whole. Systems containing living (i.e., biotic) and non-living (i.e., abiotic) components constitute biosystems, and range from genetic systems to ecosystems, all of them influencing their function and homeostasis. This multifactorial biosystem has a major effect in health, resulting into the concept of “the ecology of medical care.” Adapted from Ref. (4).

Ecology originated in natural sciences (botany and zoology) during the late nineteenth century as the study of the interaction between plants and animals with the surrounding environment, involving interdisciplinary research of biology and earth sciences. Early in the twentieth century, social scientists applied ecological principles to study human behavior and community organization (2). The word ecology is derived from the Greek *oikos*, meaning “household,” and *logos*, “study.” Thus, the environmental house includes all the organisms in it and all the functional processes that make the house habitable. Ecology is literally the study of “life at home” with the emphasis on “the totality or pattern of relations between organisms and their environment” (3). Perhaps the best way to delimit modern ecology is to consider the levels of organization seen as an ecological spectrum (Figure 1) and an extended ecological hierarchy (4).

At this basic level, human ecology can be thought of as the study of the environmental conditions in which human beings developed, and humans’ relationship with the ecosystems that support them. The same principles apply to the ecology of any species, but in the case of humans, their number on the planet, their presence in almost all terrestrial ecosystems, and their impact are largely the product of the human capacity for culture (5). The term “ecosphere” was sometimes used as an alternative for “biosphere,” thus considering the physical and biological aspects of the Earth as a unit, rather than biology alone (6).

Analysis of the environmental influence on chronic diseases has suffered continuous transformations. Concern about the environment’s effects on health has caught fire with joint health, urban-planning conferences and strategy sessions, pending legislation, and more new scientific studies (7). A framework for thinking about the organization of health care, medical education, and research has been provided that goes beyond the human ecology definition (8). This has resulted in a definition involving the relationships between people and their health care environment known as the “ecology of medical care” (4, 8, 9).

Over the years, a number of trans-National Institutes of Health (NIH) committees and NIH-supported workshops have examined the impact of the environment on autoimmune disease (AD) (10). In 2012, the National Institute of Environmental Health Sciences Expert Panel Workshop reviewed approaches for defining environmentally associated ADs in three contexts (11): (a) identifying the necessary and sufficient data to define environmental risk factors for ADs meeting current classification criteria; (b) establishing the existence of and criteria for new environmentally associated ADs that do not meet current disease classification criteria; and (c) identifying specific environmental agents that induce AD in individuals, typically in a clinical setting. They emphasized that additional efforts in all these areas are needed to achieve true consensus in this field and to define classification criteria that can distinguish environmentally associated AD cases from others with high sensitivity and specificity. Panel findings on studies of the role of environmental factors and development of ADs are shown in Table 1 (10).

**Table 1:** **Findings on human studies on the role of environmental factors and development of autoimmune diseases**.

EPW is confident of the following	EPW consider the following likely, but requiring confirmation	Broad themes to be pursued in future investigations
**Chemicals**		
Crystalline silica (quartz) contributes to development of several ADs, including RA, SSc, SLE, and ANCA-related vasculitis	Solvents contribute to development of MS	There is insufficient evidence on the role of metals, including those associated with animal models of autoimmunity, e.g., mercury
Solvents contribute to development of SSc	Smoking contributes to development of seronegative RA, MS, SLE, HT, GD, and CD	The identification of single causal agents within groups of exposures is needed (e.g., specific solvents or pesticides contributing to increased risk for the group)
Smoking contributes to development of ACPA-positive and RF-positive RA (with an interaction with the shared epitope genetic susceptibility factor)	Current smoking protects against development of UC	Studies are needed on plasticizers (e.g., phthalates and bisphenol A), some of which may be endocrine or immune disruptors, and have been associated with other immune-mediated diseases
		There is insufficient evidence on the role of cosmetics in ADs
**Physical factors**		
An inverse association exists between increased UV radiation exposure and risk of developing MS	Ionizing radiation contributes to development of HT and GD	There is insufficient evidence on a possible protective role of UV radiation on T1D
		Prospective data are needed on sun exposure as a risk factor for SLE (prior to early clinical symptoms) and dermatomyositis
**Biologic agents**		
Ingestion of gluten contributes to development of GSE	EBV infection contributes to MS development	Studies are needed on MS and vitamin D in racial/ethnic groups with darker skin (associated with UV-associated vitamin D deficiency), and examining dose-effects
		Prospective data are needed on vitamin D and other ADs
Ingestion of certain lots of l-tryptophan contributes to development of eosinophilia myalgia syndrome	Early introduction of complex foods contributes to development of T1DM and GSE	Additional studies are needed on associations of food chemicals, dyes, or additives
Dietary intake of DEPAP and oleic anilide-contaminated rapeseed oil contributes to development of toxic oil syndrome	Low dietary vitamin D intake and blood levels contribute to development of MS	Prospective studies are needed on nitrates/nitrosamines and T1DM

*Modified from Ref. (10)*.

*EPW, expert panel workshop; ADs, autoimmune diseases; RA, rheumatoid arthritis; SSc, systemic sclerosis; SLE, systemic lupus erythematosus; ANCA, anti-neutrophil cytoplasmic antibody; ACPA, anticitrullinated protein antibody; RF, rheumatoid factor; MS, multiple sclerosis; HT, hashimoto’s thyroiditis; GD, graves’ disease; CrD, crohn’s disease; UC, ulcerative colitis; T1DM, type 1 diabetes mellitus; GSE, gluten-sensitive enteropathy; DEPAP, 1,2-di-oleyl ester; EBV, Epstein–Barr virus; UV, ultraviolet*.

For many perhaps most traits, the interaction with genes and environmental factors might be genetically programed or may be purely stochastic (12). However, recent evidence pointed out that environment, more than genetics, shapes immune system (13). As a corollary, just as multiple genetic risk factors influence the development of a given AD, multiple environmental factors are also required at different periods in life, or in a specific temporal sequence to affect the immune perturbations that result in AD (4). Thus, the autoimmune ecology (AE) refers to the analysis and study of interactions among individuals and their environment leading to a break of immune tolerance and, therefore, to the development of one or some ADs in such individual. It is also likely that diverse ADs may result by the interaction of multiple environmental factors. This supports the fact that AE is a crucial component of the autoimmune tautology and reinforces the theory that ADs share several common mechanisms (14).

The AE is akin to exposome, that is all the exposures – internal (such as the microbiome, described elsewhere in this review) and external – across the lifespan, including physical environment (e.g., occupational hazards, exposure to industrial and household pollutants, water quality, climate, altitude, air pollution, and living conditions and lifestyle and behavior such as diet, physical activity, cultural practices, use of addictive substances, and so on), interacting with hereditary factors (both genetics and epigenetics) to favor or protect against autoimmunity. However, the concept of AE as well as exposome extends beyond these factors to include social factors, such as socioeconomic status (SES), quality of housing, neighborhood, social relationships, access to services, and so on. (15–17).

**Figure 2:**
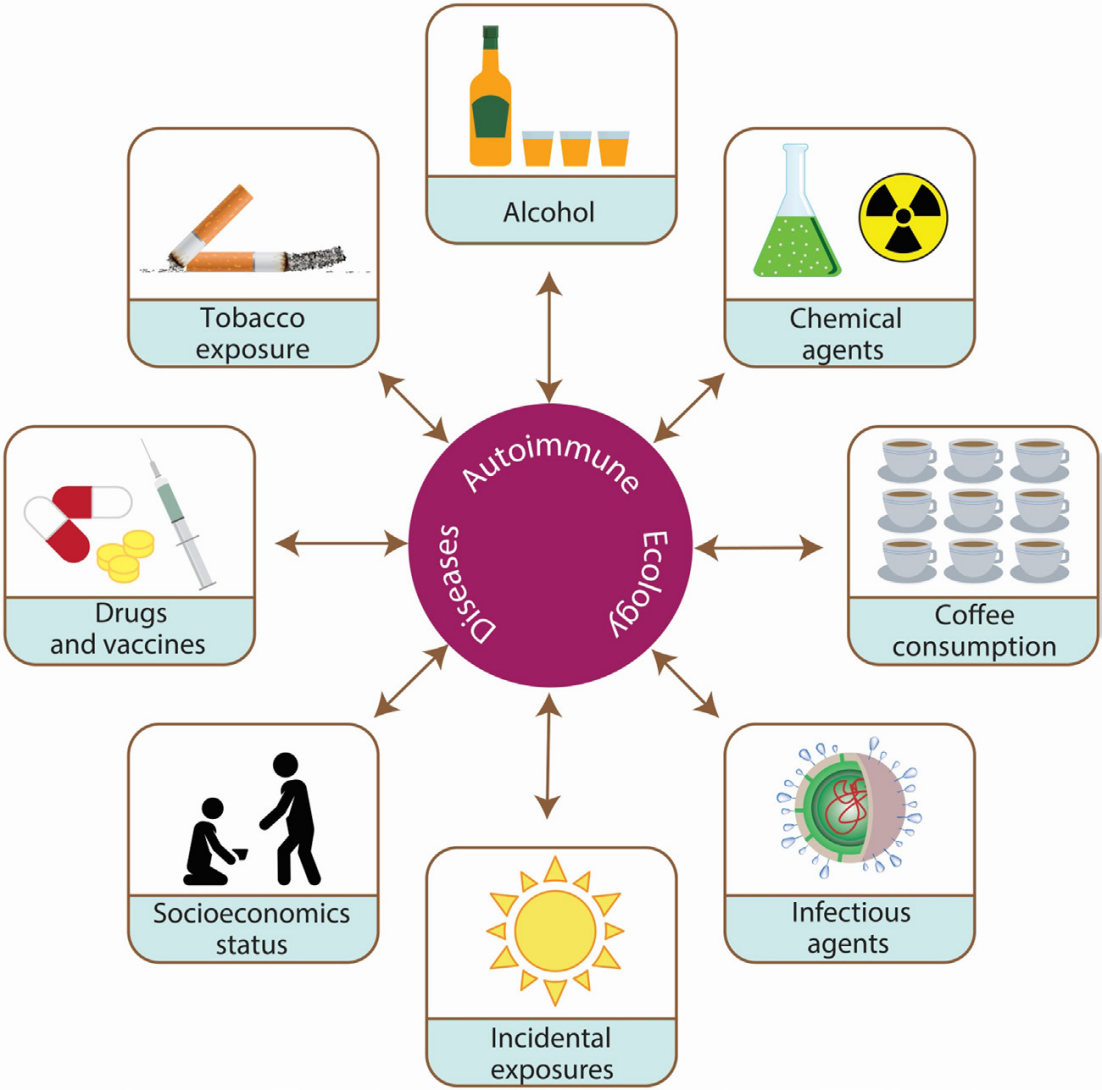
**Environmental exposures associated with the development of autoimmune diseases (ADs)**. ADs are the result of the interaction between hereditary (i.e., genetic and epigenetic) and environmental factors over time. Autoimmune ecology is defined as the environmental exposures (i.e., cigarette smoking, coffee and alcohol intake, socioeconomic status, chemical agents, vaccines, incidental exposures, infectious agents, and microbiota) influencing the development of ADs and their outcome.

To further contribute to the evidence about the nurture influence on ADs, herein we provide an overview of the AE. The focus will be on specific environmental factors and associated mechanisms that may be contributing to ADs. First is a review of the immunological and genetic bases of AE, then a review of the epidemiological bases of AE, which are mainly the influence of microbiota, lifestyle habits (i.e., alcohol, cigarette, and coffee), sex hormones, organic solvents (OSs), vaccines, SES on ADs, and environmental influence on polyautoimmunity (PolyA) (Figure 2).

## Immunological and Genetic Bases of Autoimmune Ecology

Autoimmune disease is characterized by an immune response against self-antigens affecting about 5% of the population (18). Complex diseases have multifactorial origins, which imply that combinations of the many factors involved in autoimmunity produce varying and unique clinical pictures. Gene involvement in AD is inarguable with hundreds of risk loci identified for and shared between different diseases (19). Despite the genetic associations for each distinct AD, much of the heritability remains unaccounted for (20). The heritability of ADs is not fully understood. This establishes that the missing heritability probably reflects the fact that genes do not operate alone, but in the context of the environment leading to the gene–environment interplay (21, 22). That is, the degree of disease risk contributed by one factor will be influenced by the presence or absence of another (23).

Current classification criteria have primarily relied on epidemiological comparisons of disease incidence or prevalence in exposed and unexposed cohorts. However, other approaches have included immunological and animal model studies, which added considerable supporting evidence (24). A challenge in this field has been to clarify how much evidence is required to define a given exposure as a risk factor for ADs (25). Traditional epidemiological approaches were established for a number of associations initially outlined in 1965 (26).

The environmental risk factors associated with ADs are varied as are the underlying immune mechanisms that lead to these disorders (Table 2). The following sub-topics will be discussed: effects on innate immunity, such as toll-like receptor (TLR) activation by xenobiotics, adjuvant effects, and inflammatory responses; B-cell activation; direct effect impairing the immune function, such as T helper 17 (Th17) and T regulatory cells (Treg); post-translational modifications (PTMs) of self-antigens, and epigenetic modifications, mainly DNA methylation.

**Table 2:** **Environmental factors and mechanisms involved in autoreactivity**.

Environmental factor	Main possible mechanisms
Infectious agents (bacteria/viruses)	Innate immune activation via TLRs
Adjuvants	Innate immune activation via TLRs
Sex hormones	Autoreactive B cells
TCDD/Mercury/Silver	Autoreactive B cells
Cigarette smoke	T cell impairment (Th17/Treg cells)
	Modification of self antigens
UV light	Autoreactive B cells
	Epigenetic changes (DNA methylation)

*TCDD, 2,3,7,8-tetrachlorodibenzo-p-dioxin; TLRs, toll-like receptors; Th, T helper cells; UV, ultraviolet*.

### Innate Immunity

#### Toll-Like Receptor Activation, Adjuvant Effects, and Inflammatory Responses

Toll-like receptors are membrane bound proteins present in several immune cells, including B-lymphocytes, selective populations of T cells, dendritic cells (DCs), and macrophages as well as non-immune cells, such as epithelial cells and fibroblasts. TLRs recognize invading organisms bearing pathogen-associated molecular patterns (PAMPs) and damage-associated molecular patterns (DAMPs) (27). PAMPs are conserved molecules derived from microorganisms, such as lipopolysaccharide (LPS), peptidoglycan, flagellin, and microbial nucleic acids, while most DAMPs are endogenous molecules released from dying host cell molecules upon cellular stress or tissue damage, such as oxidative stress and heat shock proteins (28). Activation of TLRs by PAMPs or DAMPs can upregulate inflammatory cytokines and chemokines and engage intracellular signaling pathways to regulate the nature, magnitude, or duration of the host’s inflammatory response (29). TLR expression may rapidly change in presence of cytokines, pathogens, and environmental factors (30). TLRs can be distinguished by their location; TLR1, TLR2, TLR4, TLR5, and TLR6 are present at the cell membrane, while TLR3, TLR7, TLR8, and TLR9 are in cell compartments, such as endosomes (31). There is constant interplay between the innate and adaptive arms of the immune system, and TLRs play a key role in this interaction.

**Figure 3:**
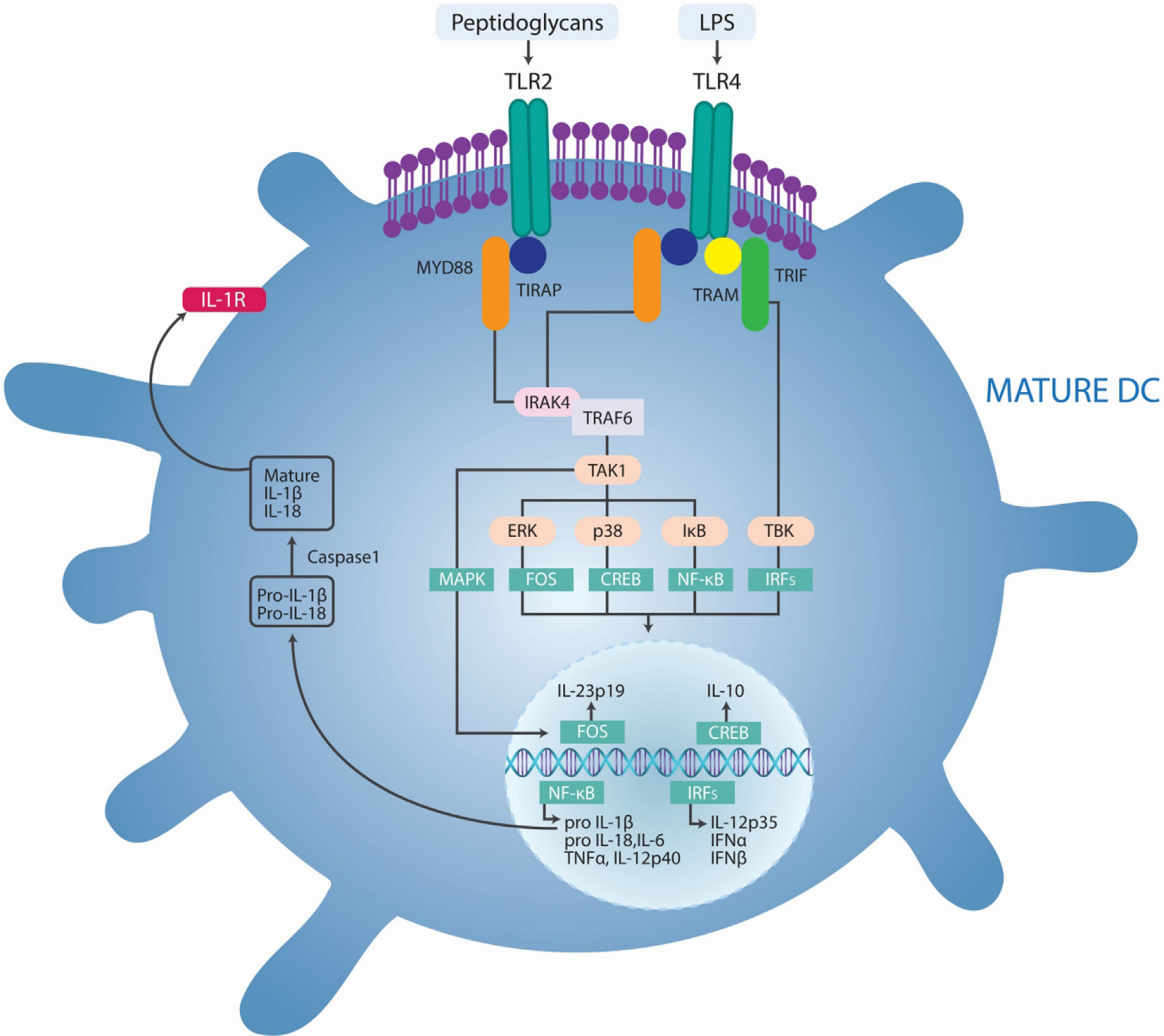
**Pathogens-induced TLR signaling cascade in innate immune cells may exacerbate autoimmune diseases**. Lipopolysaccharide (LPS) binds to TLR4 and peptidoglycans bind to TLR2, which initiates signaling by recruiting the adaptor proteins myeloid differentiation primary response protein 88 (MYD88), the TIR domain-containing adaptor protein (TIRAP), TIR domain-containing adaptor protein inducing IFN-β (TRIF), and TRIF-related adaptor molecule (TRAM). MYD88 associates with IL-1R-associated kinase 1 (IRAK1) and IRAK4 and recruits TNFR-associated factor 6 (TRAF6). This complex recruits TGFβ-activated kinase 1 (TAK1), leading to phosphorylation of NF-κB inhibitor (IκB), activation of nuclear factor-κB (NF-κB), and consequent transcription of a range of genes coding for proinflammatory cytokines, including tumor necrosis factor (TNF), interleukin-6 (IL-6), pro-IL-1β, and pro-IL-18. In addition, TLR agonists activate the interferon regulatory factor (IRF) pathways, leading to the production of IL-12p35 and type I IFNs. TLR-induced activation of TAK1 also results in phosphorylation of mitogen-activated protein kinases (MAPKs), including p38 and extracellular signal-regulated kinase (ERK). Phosphorylation of ERK promotes the transcription of the gene encoding IL-23p19 and subsequent IL-23 synthesis. Phosphorylation of p38 activates cAMP response element-binding protein (CREB), leading to IL-10 production. IL-1β activates its own receptor, which also signals via the same TLR pathway components to produce IL-1β. Adapted from Ref. (35).

The involvement of microbial PAMPs and endogenous ligands in autoimmunity through the activation of TLR and/or their increased expression acting in synergy with the formation of auto-antigen–autoantibody immune complexes has been hypothesized (Table 3) (32). High mobility group box 1 (HMGB1) is a protein that acts as a DAMP when is located outside the cell, promoting the pathogenesis of autoimmune diseases. HMGB1 can interact with multiple receptors; in particular, the binding to TLR4 can lead to the activation of NF-κB and production of IL-6 and tumor necrosis factor (TNF)-α by macrophages (33). Furthermore, HMGB1 can bind to PAMPS, such as LPS, double-stranded RNA or DNA, and act through the partner’s receptor. This synergism could induce expression of mediators, such as the prostaglandins, to promote pain and inflammation in arthritis (34). The presence of PAMPs in tissues following infection has been associated with autoimmunity. These observations provide indirect evidence that PAMPs may promote autoimmune and chronic inflammatory conditions (35). There is a well-established link between infection and ADs in both clinical settings and animal models. This link has been attributed to either molecular mimicry between pathogen-derived antigens and self-antigens or non-specific activation of innate immunity leading to a breakdown in immunological tolerance and development of self-antigen-specific T cell and antibody responses (35). Several studies in patients with multiple sclerosis (MS) have suggested that the disease may be triggered or exacerbated by infections with pathogens such as *Chlamydia pneumoniae*, *human herpes virus 6*, and *Epstein–Barr virus* (EBV) (36). *Parvovirus B19* is considered one triggering factor for rheumatoid arthritis (RA) (37). The virus was detected by PCR in synovial biopsies from 75% of RA patients compared to 17% of patients with osteoarthritis and other arthritides. Furthermore, EBV DNA and RNA were detected in 34% of RA patients with the shared HLA-DR4 epitope compared with 10% of healthy individuals (38). Infection with *Streptococcus pneumoniae* 7 days after the induction of experimental autoimmune encephalomyelitis (EAE) exacerbates autoimmunity in wild type but not *Tlr2*−*/*−** mice (39). This suggests that pathogens may exacerbate ADs via the activation of TLRs (Figure 3). Furthermore, PAMPs are present in the diseased tissues of patients with ADs. For example, peptidoglycans, which can act as ligands for nod-like receptors (NLRs) and TLR2, have been found in various cells and tissues, including in synovial tissue macrophages and DCs isolated from patients with RA (39, 40). Immunization of mice with myelin-derived peptides in complete Freund’s adjuvant (CFA) induces active EAE. CFA contains killed *Mycobacterium tuberculosis*, and PAMPs from these bacteria activate innate immune responses, which, in turn, promote pathogenic autoreactive T cell responses. Marta et al. showed that mice deficient in the adaptor protein MYD88 are resistant to EAE (41). This was associated with reduced IL-6 and IL-23 production by DCs and reduced IL-17 and interferon (IFN)-γ production by T cells. This suggests that innate immune responses initiated through TLR or IL-1R signaling are required for the induction of experimental autoimmunity. In the collagen-induced arthritis (CIA) model, which is induced by immunization with collagen and CFA, *M. tuberculosis* in CFA provides a source of PAMPs. Moreover, zymosan, a polysaccharide from the cell wall of *Saccharomyces cerevisiae* that binds TLR2, has been used to induce experimental arthritis in mice. Zymosan-induced arthritis was found to be dependent on TLR2 activation as disease was substantially attenuated in *Tlr2*−*/*−** mice (42). In addition, injection of immunostimulatory DNA sequences into joints of rats promoted development of adjuvant arthritis (43). This suggests that activation of TLR9 may also precipitate the innate immune responses that drive inflammation in joints (35).

**Table 3:** **Effects of ligands of TLR2 or TLR4 on different cells in autoimmune diseases**.

Disease	Cells	Ligands	Effects
RA	Macrophage	LPS	Enhancing the production of proinflammatory cytokines
SLE	PBMC	LPS	Dysregulation of cytokines and autoantibodies production
SSc	DCs	LPS	Secretion of cytokines
SS	Fibroblasts	LPS	Upregulation of the production of profibrotic and proangiogenic chemokines
	Human salivary cells	Peptidoglycan/LPS	Stimulating CD54 expression and IL-6 production
	PBMC	LPS/Zymosan A	Inducing the production of IL-23/IL-17

*RA, rheumatoid arthritis; LPS, lipopolysaccharide; SLE, systemic lupus erythematosus; PBMC, peripheral blood mononuclear cells; SSc, systemic sclerosis; DCs, dendritic cells; SS, Sjögren’s syndrome. Adapted from Ref. (29)*.

**Figure 4:**
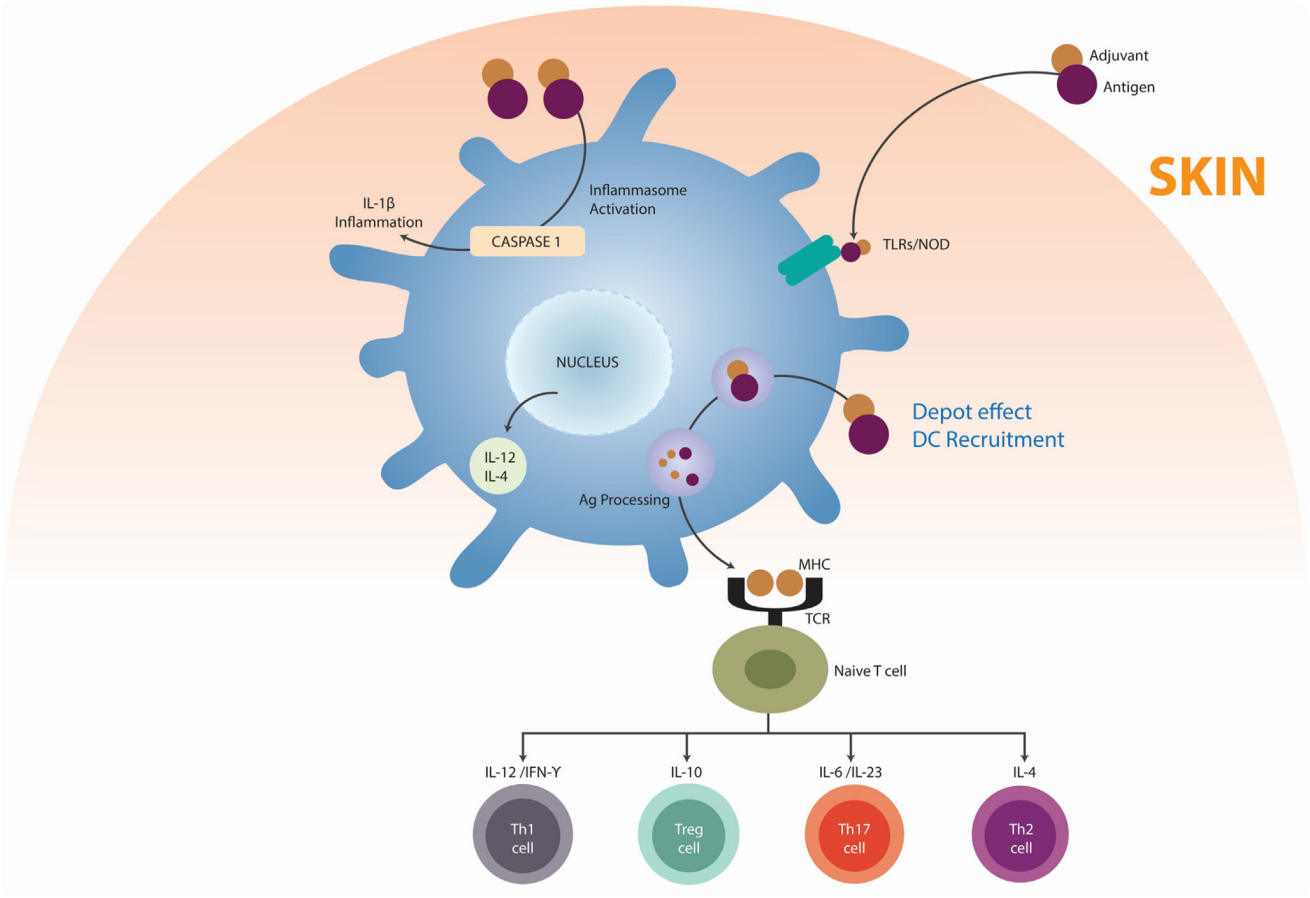
**Mechanisms by which adjuvants trigger autoimmunity**. Adjuvants (mainly alum) may function as delivery systems by generating depots that trap antigens at the injection site, providing slow release in order to continue the stimulation of the immune system, thus enhancing the antigen persistence and increasing recruitment and activation of dendritic cells (DC) (depot’s effect). Other adjuvants, essentially ligands for pattern recognition receptors (PRR), act by inducing the innate immunity by targeting the DC via toll-like receptors (TLRs). Adjuvants can direct support antigen presentation by the major histocompatibility complexes (MHC), inducing the differentiation of a naïve T cell in T helper 1 cells (Th1), T helper 2 cells (Th2), T regulatory cells (Treg), and T helper 17 cells (Th17). Adapted from Ref. (45).

Shoenfeld and Agmon-Levin coined the term “Autoimmune/inflammatory Syndrome Induced by Adjuvants” (ASIA) (44). This syndrome is characterized by non-specific and specific manifestations of AD. An adjuvant is any substance that accelerates, prolongs, or enhances antigen-specific immune response. It may stimulate the immune system and increase the response to a vaccine, without having any specific antigenic effect. Activation of the immune system by adjuvants, a desirable effect, could trigger manifestations of autoimmunity. The main substances associated with ASIA are squalene, aluminum hydroxide, silicone, mineral oil, guaiacol, and iodine gadital (45). Alum adjuvants are humoral immune potentiators in vaccine formulations. This property has been attributed to NLRP3 inflammasome activation, an intracellular multiprotein complex that mediates caspase-1 cleavage of the inactive precursor of the proinflammatory cytokine IL-1β, leading to the release of its mature form. Inflammasome-mediated cleavage of pro-IL-1β *in vitro* depends on signals that activate both TLR and nucleotide oligomerization domain-like receptors, such as NLRP3 (46, 47). The adjuvanticity of aluminum compounds is related to their association with uric acid. Alum appears to promote an inflammatory response that results in the release of uric acid from necrotic cells. Uric acid, in turn, is thought to increase the adjuvanticity of alum with an increase in IL-4 levels (45, 48). IL-4 drives the upregulation of monocytic cell surface major histocompatibility complex (MHC) class II, a crucial component in developing innate immunity. Another danger signal hypothesized to enhance the adjuvanticity of alum is host cell DNA that is released from necrotic cells (49, 50). In susceptible individuals, aluminum-based adjuvants can induce AD although this is rare. ADs correlating with alum-based vaccinations encompass conditions, such as RA, type 1 diabetes mellitus (T1DM), MS, and systemic lupus erythematosus (SLE) (Figure 4) (45).

Subcutaneous injection of mineral oil has been shown to promote anti-chromatin/DNA autoantibody production even more efficiently than squalene or incomplete Freund adjuvant. This suggests that different types of autoantibodies could be produced in response to different hydrocarbons (51). Moreover, experimental models demonstrated that adjuvant oils induced T-cell-dependent polyarthritis (52).

Silicones are a family of synthetic polymers sharing a silicon oxygen chain with varying organic side groups. The link between silicone and immune-mediated diseases has been reported in the past and is one of the cornerstones of ASIA (53). Silicone is a component of breast implants. Although there is no clear association between silicone and overt ADs (45), patients with severe immune-mediated reactions to implanted silicone devices were found to have increased immunoglobulin (IgG) in the surrounding tissue and higher levels of anti-silicone antibodies compared to asymptomatic implanted patients (54). An adjuvant action linking breast implants with development of autoantibodies has been hypothesized (55). Murine CIA model and the MRL model of murine lupus showed that silicone was responsible for increased circulating levels of IL-2 in both models as well as the production of anti-DNA autoantibodies in the MRL model. Also, in the CIA model, long-term (12 months) silicone implantation resulted in an increased incidence and severity of arthritis (56, 57). Nonetheless, most of these reports indicate that silicone implants may cause a non-specific foreign body reaction, sometimes with autoantibodies.

**Figure 5:**
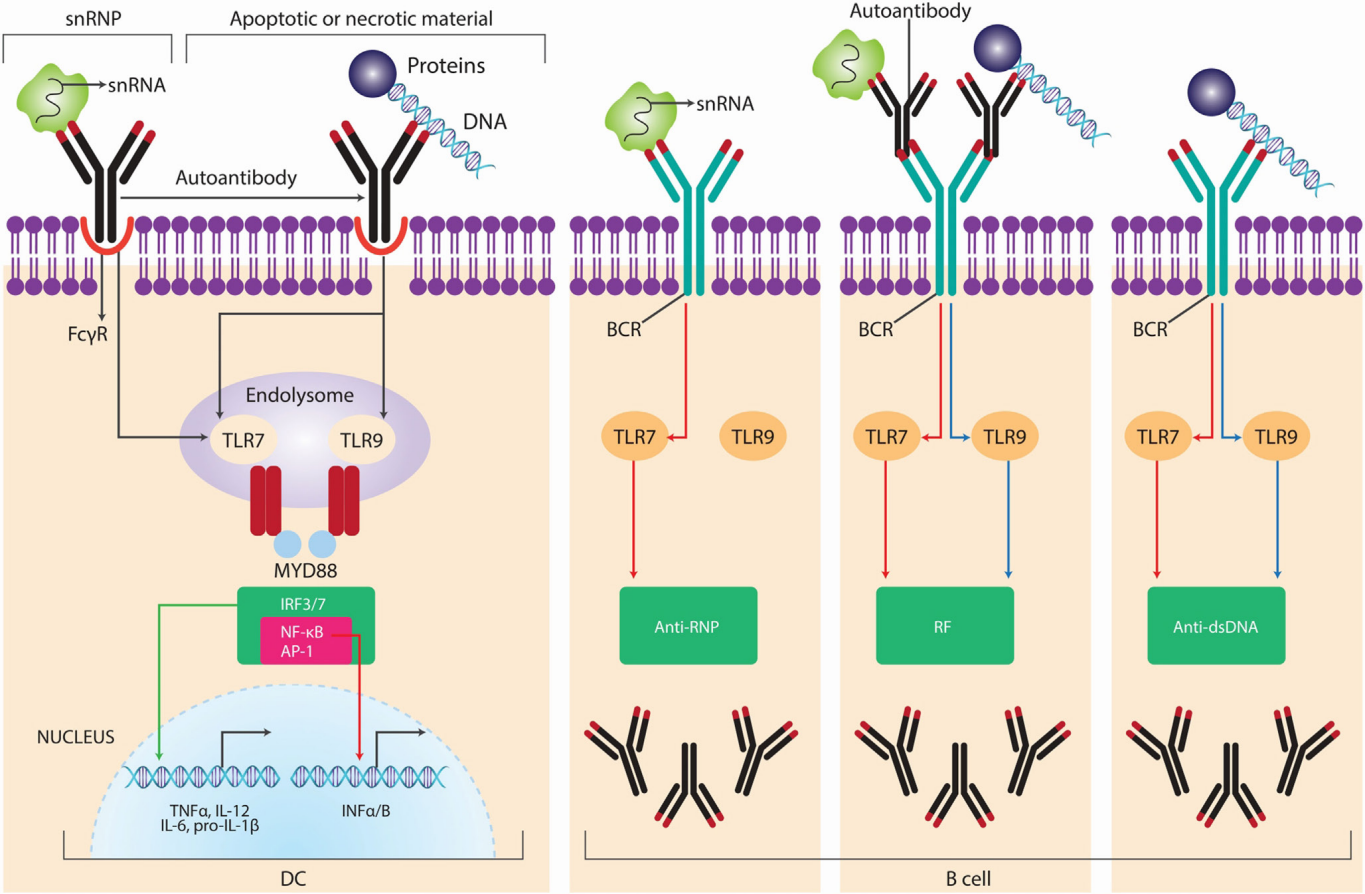
**Self nucleic acids recognition by TLRs in autoimmunity**. Autoantibodies and autoreactive B-cell receptor BCRs, such as rheumatoid factor (RF) and anti-small nuclear ribonucleoprotein (snRNP) or anti-DNA antibodies mediate the access of self-nucleic acids to endolysosomal toll-like receptors (TLRs), leading to the production of type I interferon (IFN) from dendritic cells (DCs) and autoantibodies of corresponding specificities from B cells. The major snRNP antigen for anti-snRNP autoantibodies contains small nuclear RNA (snRNA), whereas the antigenic targets of anti-DNA autoantibodies are apoptotic or necrotic material containing DNA; accordingly, anti-RNP B cells are activated by TLR7 ligands, whereas anti-DNA or RF-producing B cells are activated by either TLR9 or TLR7 ligands. Adapted from Ref. (62).

Damage-associated molecular patterns or alarmins, which are released from dead or dying cells, can also stimulate innate immune responses that lead to autoimmunity. Ultraviolet (UV) radiation promotes necrotic cells that induce danger signals or DAMPs, thus activating TLR4. This activation may be responsible for autoimmune responses (58). Furthermore, TLR4 can bind other endogenous ligands released from damaged cells, including several extracellular matrix components, such as hyaluronic acid oligosaccharides, fibronectin extra domain A, and fibrinogen, which are able to promote the synthesis of chemokines by macrophages. Also the abnormal internalization and transport of double-strain (ds) DNA fragments from necrotic cells into endosomes could induce autoimmune responses after binding to TLR3 (31). A characteristic of SLE is the presence of nuclear debris from the impaired clearance of apoptotic cells that become self-antigens able to bind B-cell receptors, induce TLR9 expression, and activate both TLR9 and B-lymphocytes with the subsequent production of autoantibodies (59). Patients showing increased apoptosis causing the release of DNA and RNA can be sensed by TLR9 and TLR7, respectively (60). Human synovial membrane cultures from patients with RA were found to express TLR2 and TLR4 and to release endogenous TLR ligands that may contribute to destructive inflammation in these patients’ joints. Another component with a role in this disease is heterogeneous nuclear ribonucleoprotein (hnRNP), an RNA- and DNA-binding complex that is a target of most nuclear antigen-specific B and T cells in systemic ADs, such as human RA and pristane-induced arthritis in rats. Moreover, hnRNP triggers TLR7- and TLR9-mediated activation of arthritogenic DCs, which activate pathogenic T cells (Figure 5) (61, 62).

### B-Cell Activation

A major role of cells in the B-cell lineage is to generate antibody-secreting plasma cells and memory B cells with an enhanced ability to respond to the specific initiating antigen. B cells increasingly emerge as part of a tightly regulated immune activation process with numerous intimate interactions with other immunocompetent cells that have been identified (63). Under normal resting conditions, B cells follow a tightly regulated life cycle with a large number of checkpoints at different stages (64). Dysfunctions at these tolerance checkpoints have been directly correlated with ADs in murine models (65, 66). In the bone marrow, B cells develop from stem cells through a series of precursor stages. During them, they rearrange their variable Ig genes to generate a wide range of unique antigen-binding specificities. B cells are critical for promoting ADs, including SLE (67–69), RA (70), T1DM (71), and MS (72), among others. A unique challenge to the maintenance of self-tolerance in the B-cell compartment is the BCR diversification within B cells that are recruited into T-dependent immune responses and ultimately enter the germinal center (GC) reaction. Somatic hypermutation (SHM) of the IgG variable region genes of GC B cells results in the occasional generation of clones with increased affinity for foreign antigen, these cells being specifically perpetuated and subsequently differentiating into the high-affinity plasma cells and memory B cells that provide long-term immunity. However, the random nature of the SHM process inevitably leads to the generation of self-reactive B cells in the GC that, unless somehow inactivated, have the potential to initiate autoantibody production. The fact that most pathogenic autoantibodies show the hallmarks of SHM and selection strongly suggests that failure to enforce self-tolerance in GCs may contribute to many autoimmune diseases. However, the self-reactive GC B cells are normally kept in check and are only rarely allowed to differentiate into autoantibody-producing plasma cells (73). B cells facilitate autoimmunity by not only secreting autoantibodies but also presenting auto-antigens to T cells and secreting proinflammatory cytokines (74, 75). Consequently, targeting B cells has become one of the most effective treatments for ADs (76). Given the biology of B-lymphocytes, certain environmental agents with the following effects are expected to have the potential to enhance B-cell-mediated autoimmunity: (a) agents that induce activation or expansion of autoreactive B cells, such as those that cause enhanced transcription of pro-survival molecules like B-cell activating factor (BAFF) and relaxed negative selection against autoreactive lymphocytes; (b) agents that might induce secretion of pathogenic antibodies, such as those that promote necrosis or apoptosis that may lead to the inappropriate presentation of nuclear components to lymphocytes; and (c) agents that induce secretion of proinflammatory cytokines as endocrine disruptors (66).

Sex hormones, such as estrogen and prolactin, can differentially activate autoreactive B-cell populations from different subsets, e.g., B2 cells, which are responsible for most of the high-affinity pathogenic antibody production and marginal zone autoreactive B cells. Therefore, a possible connection between B-cell-mediated and endocrine disruptors (i.e., environmental estrogens) has been proposed (77). dnMEK + CD2rtTA + (C57BL/6 × SJL)F1 hybrid animals disclose an impaired extracellular signal-regulated kinase (ERK) resulting in increased IgG anti-dsDNA antibody in female double transgenic mice. Moreover, male mice failed to make anti-dsDNA antibody even when both transgenes were present. Therefore, the failure in males to produce pathogenic antibody must result from sex hormones or other gender-specific differences (77). Sex hormones are thought to be responsible for the female predominance observed in SLE. The role of estrogen, which influences both humoral and cellular immunity, has been widely studied, particularly in the NZB/W F1 mouse model of spontaneous SLE (78). Autoantibody formation is accelerated by estradiol treatment of the females while tamoxifen, an estrogen blocker, ameliorates disease progression. *CD40LG*, an X chromosome gene, encodes a B-cell co-stimulatory molecule transiently expressed on the surface of activated T cells and is overexpressed on T cells from women but not men with SLE (79, 80). However, the CD40–CD40 ligand interaction is crucial to the development of T-dependent immune responses. In mice, CD40L was shown to play an important role in promoting pathogenic IgG autoantibodies and kidney disease. ERK impairment has been found to result in overexpression of CD40L protein on CD4^+^ T cells in female but not male mice, and the level of CD40L protein was twice the amount in females compared to males (79, 80). Estradiol has a central role in modulating antigen presentation, enhancing B-cell responses by increasing survival of autoreactive B cells and production of proinflammatory cytokines, such as IL-1, IL-6, and TNF-α. RA patients have higher levels of estradiol compared to healthy controls. Animals with CIA show decreased disease activity during pregnancy, and estrogen treatment in non-pregnant CIA mice leads to an increase in IgG1 antibodies, which may explain remission during pregnancy. In mice, estrogen has been shown to inhibit autoreactive B-cell apoptosis thus increasing their survival and contributing to disease severity mainly via engagement of estrogen receptor alpha (81).

Another environmental factor associated with increased risk of autoimmunity is 2,3,7,8-tetrachlorodibenzo-p-dioxin (TCDD). Its effects on the immune system are primarily mediated through the aromatic hydrocarbon receptor (AhR). TCDD is released through incineration of waste, chlorine bleaching of paper, copper smelting, and wood burning. Nevertheless, nowadays the main source of TCDD is cigarette smoking. During prenatal development, TCDD-exposed mice displayed a clear enhanced autoimmune profile, including increased splenic CD5^+^ and follicular B cells, increased autoantibody production, and increased autoimmune kidney lesions (82). Similar studies have been done on the effect of mercury and silver on autoimmunity in outbred mice, showing that both were able to induce B-cell activation and anti-nucleolar autoantibody production (83). These results are also supported by human studies showing that 40% of a human population chronically exposed to relatively high levels of mercury (a gold-mining community) exhibit relatively high levels of anti-nucleolar autoantibody in their sera (84).

Chronic infection by B lymphotropic virus can prime B-cell activation, proliferation, and clonal expansion, leading to select more autoreactive B cells (85). Although the mechanisms by which these infection agents can induce autoimmunity are not fully understood, recent studies have shown an accelerated immune senescence due to chronic exposure to latent viruses. Latent cytomegalovirus (CMV) infection in the context of a chronic autoimmune response induces the recently described “chronic infection phenotype” in CD8^+^ T cells, which retains anti-infectious effector features while exhibiting autoreactive cytolytic potential (86).

### T-Cell Impairment

T-lymphocytes are the ones mainly responsible for maintaining self-tolerance, but, additionally, they actively participate in the cell and tissue damage mechanisms in autoimmune-mediated diseases. Th17 are a subset of T helper cells that participate in autoimmune conditions (87). Th17 differentiation is regulated by various cytokines and is closely related to the development of Treg. Th17 differentiation is induced by TGF-β and IL-6 in mice, and by IL-1β and IL-6 in humans (88). AhR activated by its ligand regulates Treg and Th17 cell development (89, 90). Moreover, AhR is essential for Th17 development through the interference of STAT1 activation (91). TCDD is the best-known ligand for AhR. Smokers have a higher risk of autoantibody-positive RA and a more severe disease course (92). The mechanisms by which smoking influences the development and course of RA are not fully understood (See Post-Translational Modification of Self-Antigens, Cigarette Smoking and Cigarette Smoking and PolyA), but the interaction of TCDD with AhR and the subsequently impaired function of Th17 and Treg could certainly be one. Moreover, nicotine, a major component of cigarette smoke, stimulates the α7-nicotine acetylcholine receptors that have immunomodulatory effects. These receptors are expressed on several immune cells, including monocytes, macrophages, T- and B-lymphocytes, DCs, and fibroblasts found in the inflamed synovial tissue of RA patients. In a rat adjuvant-induced arthritis (AA), a model of human RA, nicotine-pretreated arthritic rats displayed upregulation of IFN-γ as well as IL-17. Furthermore, DCs of nicotine-treated arthritic rats showed altered levels of IL-6, IL-12p35, and IL-23, which are critical for differentiating Th1 (IL-12p35) and Th17 (IL-6 and IL-23) cells (92).

Moreover, the IL-27 level was significantly lower in rats with more severe AA. Although the role of IL-27 in ADs is not fully clear, a lower level of IL-27 with the increased severity of AA by nicotine-pretreatment has been reported, assigning a regulatory role to this cytokine in AA (93). Cigarette smoking affects the cell-mediated and humoral immune responses by inducing the release of TNF-α, TNF-α receptors, IL-1, IL-6, IL-8, and granulocyte-macrophage colony-stimulating factor. However, smoking has also been associated with decreased IL-6 production through TLR2 and 9, decreased IL-10 production via TLR2 activation, and decreased IL-1β, IL-2, TNF-α, and IFN-γ production by mononuclear cells (94). The release of intracellular antigens via tissue hypoxia or toxin-mediated cellular necrosis may precipitate an immune reaction in susceptible individuals. Smoking may also augment B-cell autoreactivity and stimulate the proliferation of peripheral T-lymphocytes (95).

In addition to IL-27 effects on rats with AA exposed to nicotine, other studies provide evidence of the implication of this cytokine in ADs. *Toxoplasma gondii* triggers the local expression of IL-27, dampening Th17 immunity and inducing chronic inflammation of the CNS (96). *In vitro* experiments showed that IL-27 suppressed the differentiation of Th17 cells triggered by IL-6, TGF-β, and a STAT1-dependent pathway. Although this work clearly shows the direct effects of IL-27 on the Th17 subset *in vitro*, IL-27 might also control the Th17 subset by indirect mechanisms *in vivo*. Indeed, IL-27 combined with TGF-β promotes the generation of suppressive T cells that produce IL-10. Whether these IL-10-secreting T cells can directly control the Th17 subset has not been formally proven as yet, but these observations indicate another possible level of regulation in the activity of IL-27 on the Th17 subset (96). Considering that exaggerated immune responses are also observed in IL-27 receptor-deficient mice infected with *Leishmania donovani*, *Trypanosoma cruzi*, or after helminth challenge, these studies provide a potential mechanism for controlling Th17-driven ADs by parasitic infections (97).

6-Formylindolo (3.2-b) carbazole (FICZ), a tryptophan photoproduct, is also a high-affinity ligand of AhR. Two different high-affinity ligands for AhR activation, TCDD and FICZ, can play different roles in the differentiation of Th-cells and the pathogenesis of EAE. On the one hand, FICZ, a high-affinity “natural” AhR ligand, appears to increase only the Th17 population, and therefore, worsen the severity of EAE (89). On the other hand and paradoxically, TCDD appears to expand only the Treg population and prevent EAE (90). Thus, depending on the ligand, AhR is capable of regulating both Treg and Th17 cell differentiation and either protect or worse ADs (98). A study in NFS/*sld* mice, a model of Sjögren’s syndrome (SS), showed that neonatal exposure to TCDD causes disruption of thymus selection. This increases the production of Th1 cytokines, such as IL-2 and IFN-γ, from splenic CD4^+^ T cells and the autoantibodies relevant for SS in the sera of these mice (99). Though not directly demonstrated, changes in the thymic microenvironment in these TCDD-exposed animals could compromise Treg development facilitating autoantibody production (66).

Ultraviolet photoproducts of tryptophan, such as the high-affinity ligand FICZ, could be synthesized *in vivo* through exposure of human skin to UV light (89). UV light, in particular UV-A and UV-B, can induce disease flares in patients with SLE and trigger disease onset (100). Cellular damage in dermal cells from excessive exposure to UV-B ranges from proinflammatory apoptotic to necrotic cell death. The exposure of auto-antigens and release of proinflammatory cytokines by phagocytic cells could ignite the autoimmune response by directly stimulating autoreactive B cells or by providing lupus antigens to preexisting autoantibodies (101). Overall, necrotic cell death might be an ideal adjuvant for self-immunization in a genetically predisposed individual. Likewise, it is well established that lupus immune response is mostly antigen driven. Therefore, a constant supply of self-antigen(s) is needed, which it is likely provided by cell death. Additionally, apoptotic blebs and bodies are potential sources of lupus auto-antigens and targets for autoantibodies (100).

Silica exposure is well known to cause not only pulmonary fibrosis known as silicosis but also influence ADs, such as RA (i.e., Caplan’s syndrome), systemic sclerosis (SSc), SLE, and anti-neutrophil cytoplasmic autoantibody (ANCA)-related vasculitis/nephritis (102, 103). Chronic exposure to silica particles activates T-responder cells (Tresp) and Treg. The activated Tresp enter the peripheral CD4^+^ CD25^+^ fraction and activated Treg are lost earlier from this fraction due to apoptosis resulting in a reduced inhibitory function. Chronically activated Tresp, in turn, may express Fas-mediated apoptosis inhibitory molecules, such as soluble Fas and so survive longer (104). These fractions may contain autoreactive clones and progress to subclinical manifestation of ADs. These cellular events also increase sIL-2R, reported in ADs as a marker of chronic activation of autoreactive T cells (103).

### Post-Translational Modification of Self-Antigens

Most proteins consist of only 20 amino acids (aa), but when considering PTMs, a protein may consist of more than 140 aa. PTMs are defined as covalent modifications occurring in a specific aa in a protein, and include acetylation, lipidation, citrullination, phosphorylation, methylation, and glycosylation. A variety of chemical modifications may be present in a protein, and these modifications are time- and signal dependent. PTMs may arise either by enzymatic modification or spontaneously. Under physiological conditions, proteins are post-translationally modified to carry out a large number of cellular events, from cell signaling to DNA replication. However, an absence, deficiency, or excess of these PTMs can lead these “new” proteins to trigger autoimmunity (105).

The strongest association of an external environmental factor with RA observed so far is smoking, which contributes to local inflammation in the lungs (106). Inflammation, in turn, leads to apoptosis or necrosis of lung cells, calcium release, and activation of peptidylarginine deiminase (PAD) enzymes. PAD enzymes cause a specific PTM called citrullination. This occurs as part of normal intracellular homeostasis, but extracellular citrullination of proteins in tissues is characteristic of many if not all inflammatory conditions (107). Citrullination changes an arginine for a citrulline by deamination of the former. The basic charge conferred by arginine changes into a neutral site in the peptide, an event that may alter the tridimensional conformation of the protein (108). These citrullinated proteins may subsequently trigger an immune response by binding to HLA-DRB1 shared epitope (SE) molecules (i.e., a 5-aa sequence motif in the third allelic hypervariable region of the HLA-DRβ chain associated with RA) on DCs leading to activation of pathogenic T and B cells, and ultimately promoting anticitrullinated protein autoantibody (ACPAs) formation. Several citrullinated proteins from fibrinogen, vimentin, α-enolase, and collagen type II constitute ACPA targets. Moreover, ACPA molecules contain additional N-linked glycans, and there is strong evidence that the introduction of N-linked glycosylation sites is a result of SHM and that the presence of these unusual sugars can modulate the binding to citrullinated antigens. This thought is sustained by the high rate of non-synonymous SHM detected in ACPA sequences (109). In particular, of the mammalian PADs characterized, PAD-4 may be associated with RA as they express in affected joints (110, 111). Polymorphisms and haplotypes at *PADI4* gene influence the risk of developing RA (112). Although the association is slight, the functional effect is strong.

Klareskog et al. showed that smoking was strongly associated with increased risk of RA in those patients expressing ACPAs, a highly specific serologic RA marker (113). To further elucidate the relevance of this gene–environment interaction circle, the odds ratio (OR) of RA risk was 21 in those individuals bearing a double copy of the HLA-DRB1 SE allele compared to 6 in patients bearing only one copy (114). The presence of HLA-DRB1 SE is the strongest known genetic factor for susceptibility to RA. The possibility that other non-HLA genes may also interact with smoking to increase susceptibility to RA is supported by Mattey et al. study showing the association between smoking and disease severity in those patients bearing a polymorphism at the glutathione S-transferase (GST) M1 locus (115). GST is important in detoxifying the xenobiotic atherogen generated by smoking. Smoking showed interaction with another major susceptibility gene in conferring risk of RA, the *PTPN22* R620W allele (108).

Using lipstick and hair dye has been associated with SLE (101). Lipstick contains several chemical compounds, such as eosin and phthalates, which have been shown to induce photosensitivity and lupus flares, the production of anti-dsDNA antibodies, and progression of renal disease in NZB/W F1 mice, respectively. This is partly and potentially due to the breach of immunological tolerance by molecular mimicry (116). While the risk of inducing lupus by hair dye containing aromatic amines is theoretically present, it is largely refuted by large observational studies in human lupus (25). Mechanistically, the potential lupus-inducing effect of aromatic amines is related to the fact that the amines are metabolized by acetylation, a process that shares a pathway similar to that of hydralazine (101). Thus, among slow acetylators, the accumulation of aromatic amines might induce SLE in genetically susceptible individuals.

### Epigenetic Modifications

Epigenetics is the study of changes in gene expression due to environmental influence without alterations in the underlying genetic sequence. Epigenetic modifications are generally classified into three main groups: cytosine genomic DNA methylation, modification of various side-chain positions of histone proteins, and non-coding RNA feedback (4).

Systemic lupus erythematosus is perhaps the best-studied AD with regard to epigenetic modification. Discordance noted in early monozygotic twin studies as well as racial and geographic variations in prevalence suggest a strong interaction between the environment and underlying genomic code in SLE (117). Environmental agents, such as cigarette smoke, mercury, silica, UV light, viral infections, and medications, have been demonstrated to induce oxidative stress. This, in turn, has been shown to inhibit and/or decrease the DNA methyltransferase 1 (DNMT1) level, thus reducing DNA methylation in CD4^+^ T cells and enhancing autoimmunity (118). In fact, increasing evidence supports reactive oxygen species being not only proinflammatory byproducts of cellular responses to infectious or inflammatory stimuli but also fine-tuning regulators of autoimmune responses varying in time, location, and extent of reactive oxygen species production (119). DNA methylation refers to the methylation of cytosine bases occurring in CpG pairs to form deoxymethylcytosine (dmC) and is a repressive modification. DNA methylation serves to not only help stabilize chromatin but also silence genes inappropriate to the function of any given cell, but for which the cell expresses transcription factors that might otherwise drive gene expression (118).

**Figure 6:**
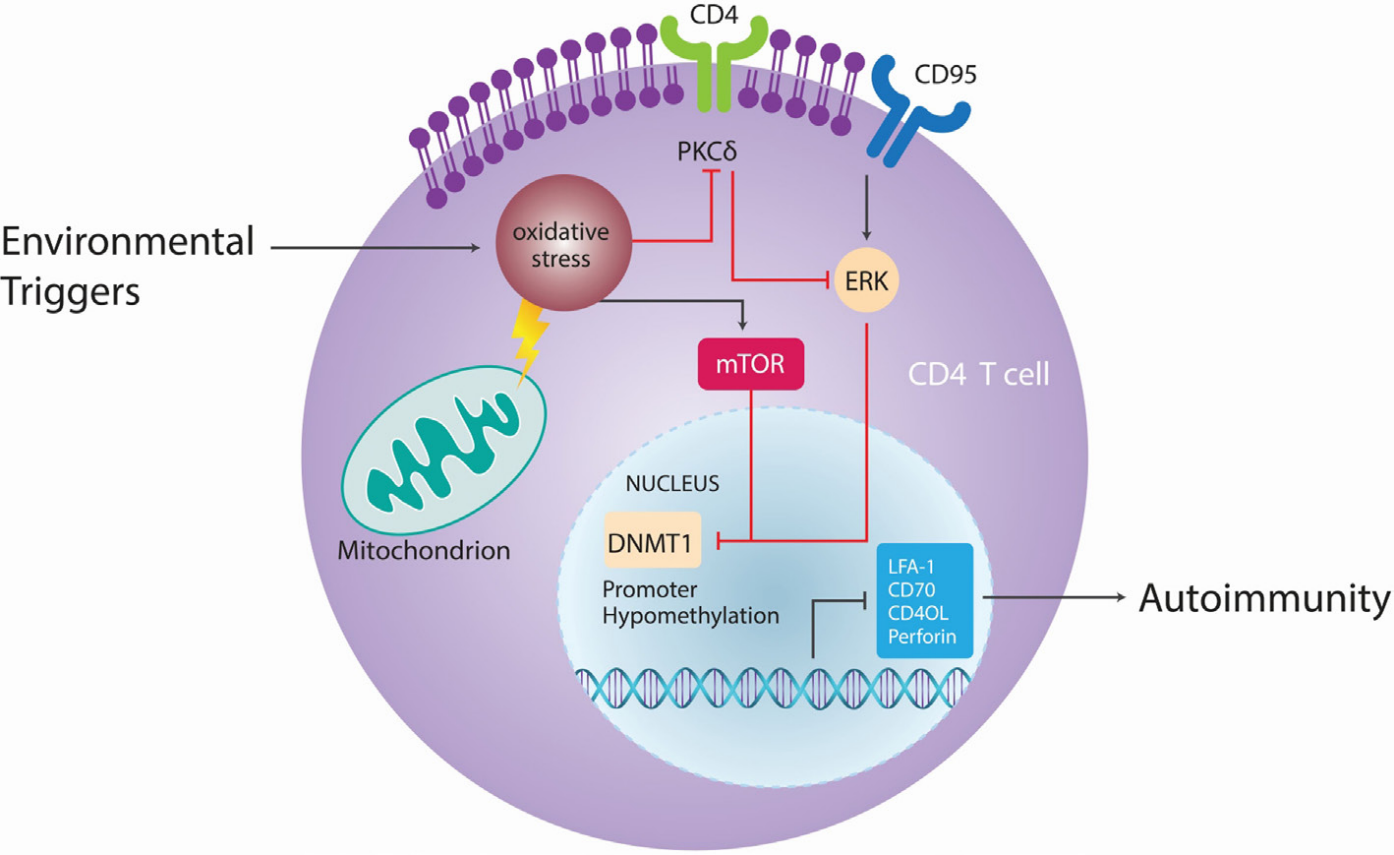
**Epigenetic autoimmune ecology pathways**. Different environmental factors, such as cigarette smoke, chemicals, UV light, and viral infections cause oxidative stress, which lead to activation of the mammalian target of rapamycin (mTOR) pathway that can directly inhibit DNA methyltransferase 1 (DNMT1). Oxidative stress contributes in CD4^+^ T cells to site-specific decrease in phosphorylation of protein kinase C (PKC)δ T^505^, leading to functional loss of PKC and parallel reduction of extracellular signal-regulated kinase (ERK) phosphorylation. As a result of ERK activity, expression of DNMT1 is reduced, DNA hypomethylation occurs and CD4^+^ T cells become autoreactive. Adapted from Ref. (101).

T cells from patients with active lupus have decreased total dmC content and decreased DNMT1 transcripts relative to patients with inactive disease and normal controls (120). During mitosis, the level of DNMT1 is regulated partly by the activated ERK signaling pathway. Patients with active lupus have impaired T cell ERK1/2 phosphorylation, and the degree of impairment is proportional to disease activity (121). The deficient ERK pathway contribution to lupus has been further supported in mouse models by the finding that inhibiting T cell ERK pathway with a MEK inhibitor could induce autoreactivity *in vitro* and lupus-like autoimmunity in mice by promoting DNA hypomethylation and overexpression of methylation-sensitive genes (122). Decreases in ERK pathway signaling are concomitant with a diminished PKCδ phosphorylation. This direct link between PKCδ and ERK suggests that abnormalities in the PKC–ERK pathway from impaired PKCδ activation may contribute to human lupus through effects on DNA methylation in T and perhaps other cells (101, 123) (Figure 6).

Demethylated, autoreactive CD4^+^ T cells overstimulate antibody production by B cells and kill macrophages, thus releasing apoptotic nuclear material that stimulates lupus-like autoantibodies (124). Moreover, in CD4^+^ T cells, ITGAL (CD11a) is an integrin involved in both costimulation and cellular adhesion that, upon dimerization with CD18, forms leukocyte function-associated antigen 1. CD11a is overexpressed in SLE patients’ CD4^+^ T cells and correlates with disease activity (125). The upstream ITGAL promoter is demethylated in active lupus patients compared to controls, the degree of demethylation correlating with disease activity. CD4^+^ T cells from patients with active lupus were also found to overexpress perforin, CD70, CD40L, and KIR due to demethylation in the respective promoters (118). CD40L is encoded on the X chromosome, so T cells from women have one methylated and one unmethylated CD40L gene, while T cells from men have only one unmethylated gene (79). Furthermore, injecting experimentally demethylated CD4^+^ T cells into syngeneic mice causes anti-DNA antibodies and a lupus-like disease (118).

After exposure to UV-B irradiation, the growth arrest and DNA damage-induced 45a GADD45a levels are increased and accompanied by high levels of CD11a and CD70. The levels of GADD45a are inversely proportional to DNA methylation levels in human lupus CD4^+^ T cells. The transfection of GADD45a *in vitro* is sufficient to increase expression of these methylation sensitive genes and induce autoreactivity (126). Furthermore, increases in oxidative stress lead to activation of the mammalian target of rapamycin (mTOR) pathway, which can directly inhibit DNMT1 (127).

The importance of epigenetics in initiating and perpetuating autoimmunity is summarized in Table 4 (117). However, there is no clear evidence of the environmental agent involved in each of the epigenetic mechanisms known for these ADs. Despite great efforts and many studies, several environmental agents have been associated with a variety of ADs, but little is known regarding the specific cellular and molecular mechanisms involved for each causal environmental agent.

**Table 4:** **DNA methylation process in autoimmune diseases**.

Disease	DNA methylation
SLE	T cells hypomethylation, IFN signature, hypomethylation of CD70, CD11a, CD40L, perforin
RA	T cells and CD40 hypomethylation, synoviocytes hypermethylation
SSc	T cells and fibroblasts hypomethylation, Wnt pathway genes hypermethylation

*RA, rheumatoid arthritis; SLE, systemic lupus erythematosus; SSc, systemic sclerosis; IFN, interferon*.

## Epidemiological Bases of Autoimmune Ecology

### Microbiota

There are different microorganisms that populate the gut known as intestinal microbiota. This process starts at the time of delivery and breastfeeding, and it plays an important role in the homeostasis of the human body. Indeed, there are microorganisms in the microbiota that produce enzymes and molecules, which are not generated by human beings. Therefore, microbiota is important to normal metabolism because it is capable of degrading the different components in food (128, 129). However, microbiota may also influence other systems that do not seem to be related to the gut, such as the immune system. Moreover, it actively participates in the education of the immune system. For example, the development of Th17 and Treg lymphocytes is highly dependent on the interactions of commensal bacteria with host cells in the intestine. That is why it is possible to establish a connection between microbiota and autoimmunity or inflammatory disorders (128–131).

Studies of germ-free (GF) animal models have demonstrated deficiencies in their immune system. It is noteworthy that microbiota is the first barrier against pathogenic microorganisms. They may produce molecules against the pathogens during infection because they occupy the same niche, thus competing for the same place. GF mice show deficiencies in T-lymphocyte differentiation within the lamina propria in the presence of IgA in mucosal layers and alterations in the homeostasis of Th populations (Th1, Th17, and Treg). Furthermore, GF mice spleens show few GCs that indicates abnormal development and maturation of cells in the lymphoid follicles (128–130). As a result, the cytokine production and the normal maturation process of immune cells are greatly affected (129).

Microbiota varies from one individual to another and even in the same individual over the course of his life. However, there are many factors that influence the composition of microbiota. Initially, newborns are sterile, and their microbiota depends on maternal transfer at the time of delivery, breastfeeding, and skin contact with the mother. For example, there are differences between children that were born by natural delivery and cesarean section, and also between children that were breastfed and those fed with formula. The latter group in both cases is colonized by potential pathogens and, in contrast with first group, they present a lack of beneficial commensal microorganisms (129, 132). Nevertheless, their own genetic background plays an important role in determining the microbiota before contact with the mother’s microbiota. For example, animal models with gene alterations related to the innate immune system have problems in the signaling pathways associated with pattern recognition receptor (PRR) recognition (e.g., TLRs and NODs). These models also have important differences in the pattern of microbiota microorganisms compared to wild-type animals (128–130).

In addition, the evolution of medical treatment, stress, and quality of life influence microbiota development. For example, antibiotics are the treatment for infectious diseases, but their use is linked with the loss of beneficial microorganisms. Moreover, they alter the microbiota ecology because they affect the equilibrium between different bacterial species in the gut, which allows opportunistic pathogens to attack the body (129). Finally, diet plays a central role in the homeostasis of the microbiota because it defines which microorganisms can survive in the gut due to differences in the preferences of microorganisms for energy sources. Thus, diet composition is extremely important in microbiota maintenance. Components from plants are the energy source for beneficial microbes and promote their growth over other microbes. It has been suggested that differences in the modern western diet could be causing the rapid increase in diseases, such as asthma (129, 133). For example, one study shows how a switching from a low fat, vegetable-rich diet to a high fat, high sugar diet could alter the microbiota within 1 day (129, 134).

**Figure 7:**
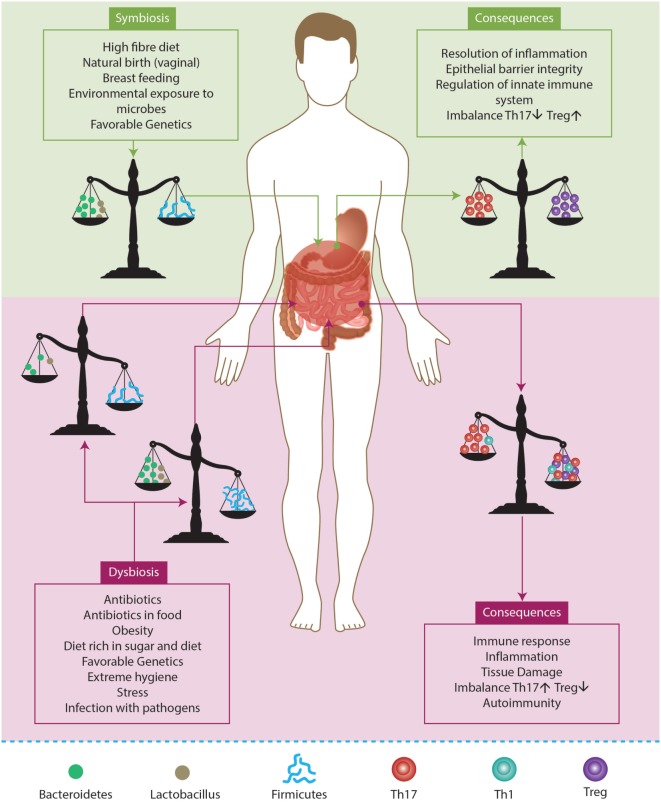
**Factors influencing gut microbiota**. A balancing microbiota in symbiosis influences the equilibrium between T regulatory cells (Treg) and effector T cells (Th1, Th17). As a consequence, the gut can respond to an infection but this also leads to the permanence of microbiota and favors the host. By contrast, if the environment induces the rupture of the equilibrium, it induces a state of dysbiosis. Hence, the percentage of anti- inflammtory microorganisms (e.g., *Bacteroidetes* and *Lactobacillus*) may be lower than proinflammatory microorganisms (e.g., *Firmicutes*). Eventually, this may lead to an inflammatory profile that favors the development of autoimmunity. Adapted from Ref. (129, 130).

Altogether, there is a lot of variability in microbiota between individuals, and it also varies based on the anatomic area of the human body (135, 136). Fluctuations in microbiota population have been described in patients with ADs (128). In addition, most of the studies have established a relationship between the microbiota inhabiting the gut and its influence on health. Usually, microorganisms that live in the gut are not pathogenic under healthy conditions, and they have a positive effect on the host (130). Nevertheless, some commensal bacteria may drive the preferential development of Treg, while others promote Th17 response and inflammation. These bacteria favor the production of regulatory molecules and cytokines, e.g., Foxp3 and IL-10, which characterize the regulatory cells, Treg in particular. Specifically, species such as *Bacteroides fragilis* and the genus *Lactobacillus* and *Bififobacterium* greatly promote the presence of Treg in the gut. By contrast, a pathogenic phenotype characterized by Th17 response and proinflammatory cytokines is promoted by segmented filamentous bacteria, such as *Firmicutes* (Figure 7). Moreover, it has been demonstrated that this kind of bacteria is able to induce the production of IgA in the small intestine. Th17 response certainly has its own positive role in the case of infection control, but it is also critical in the development of inflammatory and autoimmune diseases (128–130, 135).

Any change in microbiota could induce a dysbiosis and a pathological event. At present, the use of antibiotics, such as ampicillin, gentamicin, and vancomycin, may affect the balance toward bacteria that stimulate the Th17 response. At the same time, the consumption of a diet rich in sugar and fat favors a rise in the population of *Firmicutes* and a reduction in the *Bacteroidetes* (128, 129, 135). Finally, the influence of microbiota on the immune balance does not depend exclusively on the presence of the microorganisms, but it also depends on molecules that are produced by microbes and can stimulate specific pathways associated with immune tolerance (129). An example is the anti-inflammatory effect of the short-chain fatty acids, e.g., acetate, propionate, and butyrate. These fatty acids can bind to the G-protein-coupled receptor (GPR43 and GPR120) that is expressed in most of the innate immune system cells and, thus, produce an anti-inflammatory action (129). The anti-inflammatory events induce the maintenance of the epithelial barrier, regulate apoptosis, diminish oxidative DNA damages, and regulate cytokine production, phagocytosis, and neutrophil recruitment. At the same time, the peptidoglycan and the polysaccharide A teach the immune system to recognize potential pathogenic bacteria, and they also help in the correct development of balanced T-cell response in the gut. This balanced response promotes cell–cell interaction by modification of protein expression in immune cells (129).

Different studies have shown the microbiota as a changing ecosystem that has many factors in the model (136). Most of these studies try to determine what these microorganisms are and how they vary over time. However, the study of these microorganisms represents a challenge in terms of methodology because their identification was done by culture, and most of them cannot be grown in laboratory conditions. Therefore, most of them are now being studied with the help of DNA high-throughput analysis. Indeed, analysis of the entire DNA allows the study of all the organisms present in a sample (137, 138).

Due to the current metagenomic results (i.e., the study of the genomes in a specific environment) (139, 140), the analysis of the microbiota is moving forward and new OMICS have been identified. However, the real involvement in host systems does not depend exclusively on the presence of a specific microbiome. For instance, metagenomics would be just a representation of what these microorganisms are. Moreover, molecules produced by microorganisms can interact with the host at different levels leading to diverse outcomes. In consequence, it is important to study gene expression (metatranscriptomics), proteins (metaproteomics), and metabolites (metametabolomics) from a microbiome. These analyses, including possible interactions within the host system and under certain conditions can settle the real mechanisms by which specific microorganisms (present in a particular microbiome) may influence the host system and, thus, lead to complex disease modulation, such as ADs (138).

There are different strategies for analyzing data. Currently, the sequencing of the 16S rRNA gene has made it possible to describe prokaryote taxonomic diversity. This gene has been used for this type of analysis for three main reasons. Primarily, it is present in every population member due to its essential role in protein translation in all prokaryotes. Second, 16S rRNA always and only differs between individuals with different genomes. Third, it is considered an evolution marker between species because its function is extremely important, so any change in its sequence can be potentially lethal. Therefore, the 16S rRNA sequence analysis brings us to the construction of operational taxonomic units (OTUs) based on the percentage of similarity between sequences. Then, comparison between multiple databases leads to the identification of species within a sample. Finally, the population becomes a 16S rRNA sequence collection where the number of unique sequences represents the number of microorganisms (136).

Furthermore, high-throughput DNA sequencing technologies have recently become very useful for metagenomic analysis (i.e., pyrosequenciation). Thus, different strategies for different types of genome assembly and annotation within a sample are used to ensure an accurate description (141). In addition, potential proteins encoded in each genome can be analyzed based on DNA sequence. As a result, it is possible to assign a role or function for a specific microorganism within the microbiome based only on their genome capabilities (137).

The relationships between infection and ADs and its main mechanisms have been outlined (i.e., host–guest interaction). Nonetheless, most of the interactions and mechanisms that influence this relationship are still unknown. Microorganisms may alter and deregulate gene transcription, translation, and human metabolic processes. This means that the effect induced on the host by a microorganism is not caused by the presence of the microorganism itself but also by the metabolic and genetic polymorphism of the microorganism. In particular, intracellular pathogens may have a direct influence on gene regulation and protein expression inside host cells (131, 132, 142).

In the last few years, most of the large cohort studies have evaluated genetic factors that may predispose to ADs. In addition, expression and proteomic analysis have also been done, and most of them have tried to find a way to establish predictor genetic factors for the diseases. However, these studies do not take into consideration the DNA, RNA, and proteins from microorganisms that could be considered potential “contamination,” and which should be considered a source of information that would help complete the overall picture of molecular interactions between infection and ADs and make it understandable (132, 143, 144).

Last but not least, familial autoimmunity and polyautoimmunity should be incorporated into the study of infection and ADs (145, 146). ADs may be associated with a specific family group exposed to a particular environment. Within this environment, family members will be in contact with the same microorganism, and they will develop the same microbiota. As a new common mechanism for ADs, microbiota can be “inherited” from parent to child, and it can also be shared among siblings (135).

### Lifestyle Habits and Autoimmunity

#### Cigarette Smoking

Cigarette smoking has been widely associated with cardiovascular disease (CVD), pulmonary disease, cancer, and increased overall mortality. The association with ADs has also been extensively studied and confirmed based on the direct tissue damage and inflammatory response to tobacco (92).

For RA, a clear causal relationship between tobacco and RA risk was established in 1987 even though previous studies had already recognized its association with RA comorbidities (147–149). Since then, many case-control and cohort studies have evaluated and confirmed this relationship (150–154). Additionally, cigarette smoking was found to increase the risk of seropositive RA (rheumatoid factor (RF) and/or ACPA) both in Caucasians and Latin Americans (155–157). Tobacco also influences the phenotype or clinical expression of disease, and is related to earlier onset of disease (158, 159). For instance, smokers have a worse outcome with greater disability and disease activity, and have usually taken significantly more disease-modifying antirheumatic drugs (DMARDs) combinations or biologics than non-smokers (155, 158, 160).

A dose–response association between cigarette smoking and RA development was also confirmed by a recently published meta-analysis. Three prospective cohort studies and seven case-control studies, including 181,100 subjects and 4,552 RA cases were considered for analysis. Compared to non-smokers, patients who smoked 1–10 pack-years presented an increased risk for RA [relative risk (RR): 1.26, 95% CI: 1.14–1.39]. Those who smoked more than 20 pack-years had an even higher risk, with a RR of 1.94 (95% CI: 1.65–2.27) and, for those with more than 40 pack-years, the RR was 2.07 (95% CI: 1.15–3.73) (161).

A systematic review and meta-analysis on the influence of cigarette smoking on disease activity and joint erosions in RA showed a negative association between smoking and EULAR response (OR: 0.72, 95% CI: 0.57–0.91; *p* = 0.005) and DAS28 remission (OR: 0.78, 95% CI: 0.63–0.96, *p* = 0.023). DAS28 was significantly higher in current smokers [mean difference (MD): 0.29, 95% CI: 0.12–0.44, *p* < 0.001] as well as erosive score [standardized mean difference (SMD): 0.38, 95% CI: 0.04–0.72, *p* = 0.028]. Data were ambiguous for erosion progression during follow-up (OR: 0.93, 95% CI: 0.72–1.2; *p* = 0.59) (162).

As for RA, cigarette smoking is also associated with increased risk of SLE. A meta-analysis showed that for current smokers, compared to non-smokers, the odds for developing SLE were significantly higher (OR: 1.50, 95% CI: 1.09–2.08) (163). Additionally, current smokers with SLE are more likely to have anti-DNA antibodies than non-smokers (164). This last finding is related to the evidence of increased disease activity (i.e., SLEDAI score) observed in SLE patients that are current smokers compared to non-smokers (165). Furthermore, cigarette smoking (i.e., ever smoking) has been associated with CVD in patients with SLE (166). When smoking and coffee factors were combined, the association with cardiovascular risk in SLE remained statistically significant [adjusted odds ratio (AOR): 1.82, 95% CI: 1.05–3.13, *p* = 0,03].

Environmental exposure (i.e., ever smoke, coffee consumption, silicone implants, organic solvents, hair dye, and pesticide exposure) influences the risk of developing lupus nephritis (LN) in a Latin American population when examined by latent variable analysis using the two-parameter logistic item response theory model (167).

Exposure to tobacco smoke has also been associated with a higher incidence of other AD, such as MS. Cigarette smoking increases the risk of progressive disease from the moment of diagnosis. Also, current smokers progress from relapsing-remitting disease to secondary progressive disease faster than non-smokers (168, 169). Regarding autoimmune thyroid disease (AITD), a meta-analysis was done including eight observational studies that showed an association between Graves’ disease (GD) and current and ever-smoking status, with an OR of 3.02 (95% CI: 2.09–5.22) and 1.9 (95% CI: 1.42–2.55), respectively. An association between Hashimoto thyroiditis and tobacco was also found (OR: 1.92, 95% CI: 1.25–2.93) (170).

Furthermore, a strong association has been found between primary biliary cirrhosis (PBC) and smoking in three large case-control studies (171–173). In addition, cigarette smoke accelerates the progression of PBC (174), all of these pieces of evidence raising the issue of the influence of tobacco on Th1 response.

In conclusion, cigarette smoking is one of the best-recognized environmental factors associated with AD. Different outcomes have been related to this association: disease onset, progression, activity, and treatment response. Relevant evidence available is summarized in Table 5 (175).

**Table 5:** **Association between smoking status and autoimmune disease onset and evolution**.

Disease	Disease onset			Evolution			Reference
	Ever	Current	Past	Ever	Current	Past	
RA	POR 1.89 (1.56–2.28)^a^	POR 1.87 (1.49–2.34)^a^	POR 1.76 (1.33–2.31)^a^	NA	EULAR response POR 0.72 (0.57–0.91)	DAS28 remission POR 0.78 (0.63–0.96)	(161, 175)
	POR 1.27 (1.12–1.44)^b^	POR 1.31 (1.12–1.54)^b^	POR 1.22 (1.06–1.40)^b^				
SLE	NA	POR 1.5 (1.09–2.08)	POR 0.98 (0.75–1.27)	Mean SLEDAI 15.63 (12.96–18.3)	Anti-DNA seropositivity OR 4.0 (1.6–10.4)	Mean SLEDAI 9.64 (7.61–11.67)	(163, 164)
MS	AOR 1.32 (1.10–1.60)	NA	NA	NA	Progression to SPD HR 2.5 (1.42–4.41)	NA	(168, 169)
AITD	POR 1.90 (1.42–2.55)	POR 3.30 (2.09–5.22)	POR 1.41 (0.77– 2.58)	Development of GO POR 2.53 (1.70–3.77)	Development of GO POR 2.18 (1.51–3.14)	NA	(170)

*RA, rheumatoid arthritis; SLE, systemic lupus erythematosus; MS, multiple sclerosis; AITD, autoimmune thyroid disease; POR, pooled odds ratio; AOR, adjusted ODDs ratio; HR, Hazard ratio; NA, not available; EULAR, European League Against Rheumatism; DAS28, disease activity score-28; SLEDAI, systemic lupus erythematosus disease activity index; SPD, secondary progressive disease; GO, Graves opthalmopathy. All results in parentheses correspond to 95%CI*.

*^a^Results in men*.

*^b^Results in women*.

*Adapted with author’s permission from Ref. (4)*.

#### Alcohol Consumption

Multiple studies have evaluated the relationship between alcohol consumption and the risk of ADs, especially regarding RA and SLE. Most of the available evidence has come from case-control studies, which show a reduced risk for both RA and SLE with a low-to-moderate alcohol intake (157, 176–180). By contrast, some case-control studies (181, 182) and a prospective cohort analysis (183) have shown no association between alcohol and autoimmunity.

Since the evaluation of lifestyle impact on disease by case-control studies may present a recall bias, two prospective studies have been done on the evaluation of alcohol and RA. The first was subdivided into two large cohorts, the Nurses’ Health Study NHS and NHSII, showing 580 incident cases of RA in 1.9 million person-years from 1980 and 2008 and 323 incident cases of RA in 1.78 million person-years from 1989 to 2009. In these cohorts, the pooled adjusted hazard ratio (HR) for moderate alcohol use in seropositive RA was 0.69 (95% CI: 0.50–0.95) indicating a protective effect of alcohol consumption (184). In the second cohort study, 34,141 women were followed from 2003 to 2009 with 197 incident cases of RA identified. Here, there was a statistically significant 37% reduced risk of RA among women who drank more than four glasses of alcohol per week, compared to those who drank less than one glass per week or non-drinkers (RR: 0.63, 95% CI: 0.42–0.96) (185).

Additionally, two meta-analyses for RA and one meta-analysis for SLE have been done to evaluate risk associated with alcohol intake. For RA, one of the meta-analyses included nine studies (six case-control and three cohorts) comparing drinkers vs. non-drinkers and finding an adjusted OR of 0.78 (95% CI: 0.63–0.96). The protective effect was stronger in seropositive RA (0.52, 95% CI: 0.36–0.76) than that in seronegative RA (0.74, 95% CI: 0.53–1.05). Subgroup analysis by study design identified a significant association in case-controls but not in cohort studies (186). The second study considered eight prospective studies that contained 195,029 participants and 1,878 RA cases. Dose–response meta-analysis of the study data revealed that, compared to no-alcohol consumption, the adjusted RR was 0.93 (95% CI: 0.88–0.98) for 3 g/day of alcohol consumption, 0.86 (95% CI: 0.76–0.97) for 9 g/day, 0.88 (95% CI: 0.78–0.99) for 12 g/day, 0.91 (95% CI: 0.81–1.03) for 15 g/day, and 1.28 (95% CI: 0.94–1.73) for 30 g/day, indicating an inverse association between low-to-moderate alcohol intake and RA risk (187). For SLE, six case-control studies and one cohort study were included for analysis, and an overall significantly protective effect was found when considering SLE patients treated for <10 years (OR: 0.72, 95% CI: 0.55–0.95) (188).

Despite the evidence showing the protective effect of alcohol in two common ADs, it should be remembered that the evidence for the harmful effects of alcohol remains stronger than its benefits (181). Thus, no promotion of alcohol intake to prevent ADs is recommended.

#### Coffee Consumption

A few studies have been done to examine the effect of coffee on the risk of ADs. A meta-analysis evaluated the association between coffee or tea intake and the risk of RA. This analysis included five studies (three cohorts and two case-control) involving 1,279 cases of RA and 133,622 controls. The final effect size showed a significant association between total coffee intake and RA incidence (RR: 2.426, 95% CI: 1.060–5.554, *p* = 0.036). When stratified by seropositivity, a significant association between coffee consumption and seropositive RA incidence was found (RR: 1.329, 95% CI: 1.162–1.522), but no association in seronegative RA (RR: 1.093, 95% CI: 0.884–1.35, *p* = 0.411) (189). Another study evaluated the relationship between coffee intake and methotrexate (MTX) efficacy in RA, measured by DAS28 score, Multi-Dimensional Health Assessment Questionnaire (MDHAQ) score, and duration of morning stiffness with no statistical difference between groups (190).

Concerning SLE, a cross-sectional study was done on 310 Colombian patients to evaluate the risk factors associated with the appearance of CVD as the leading cause of mortality. The estimated AOR for coffee consumption was 1.75 (95% CI: 1.01–3.04, *p* = 0.043). Additionally, to isolate the interaction of smoking and coffee consumption, two regression models were made, showing an independent effect of coffee and tobacco on CVD. Their interaction remained significant (AOR: 1.82, 95% CI: 1.05–3.13, *p* = 0.03), thus demonstrating their synergism (166).

The association between coffee consumption and ADs has also been evaluated in latent autoimmune diabetes in adults with no statistically significant association (191). However, other studies have demonstrated the opposite relationship between maternal coffee consumption and developing advanced β-cell autoimmunity in the offspring (highest quarter vs. lowest HR: 0.62, 95% CI: 0.40–0.97, *p* = 0.04) (192).

Two studies have evaluated coffee consumption, primary sclerosing cholangitis (PSC), and PBC. The first study included data from 480 patients with PSC, 606 with PBC, and 564 healthy volunteers. Interestingly, 24% of patients with PSC had never drunk coffee compared to 16% of controls (*p* < 0.05), and only 67% were current drinkers compared to 77% of controls (*p* < 0.05). Furthermore, patients with PSC consumed fewer lifetime cups per month (45 vs. 47 for controls, *p* < 0.05) and spent a smaller percentage of their lifetime drinking coffee (46.6 vs. 66.7% for controls, *p* < 0.05). Moreover, among PSC patients with concurrent ulcerative colitis, coffee protected against proctocolectomy (HR: 0.34, *p* < 0.001). By contrast, patients with PBC and controls did not differ in coffee consumption parameters (193). The second study included 240 patients with PSC and 245 contro l subjects. The results suggested a protective effect of both cigarette smoking and coffee consumption at the age of 18 with the development of PSC (*p* < 0.05 and *p* = 0.048, respectively) (194).

### Socioeconomic Status and Autoimmunity

The social environment includes social, political, economic, and cultural norms and values that result in an unequal distribution of resources that can negatively impact health. Many studies have evaluated the association between SES and chronic diseases, showing greater morbidity and mortality in people with lower socioeconomic position (195).

**Figure 8:**
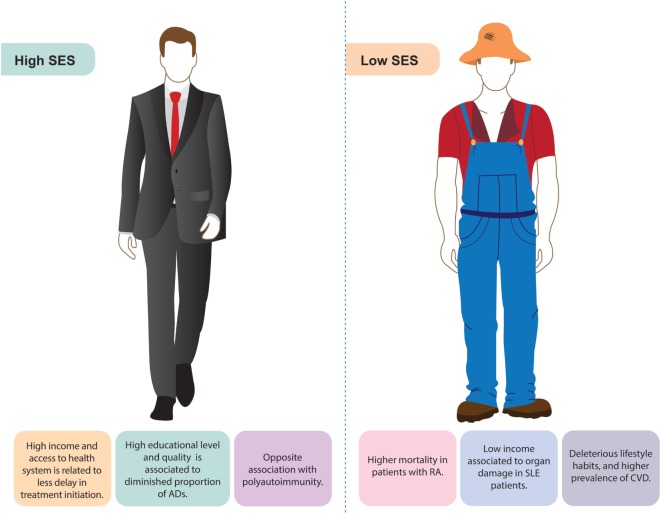
**Socioeconomic status (SES) and autoimmune diseases (ADs)**. Some of the main findings associated with autoimmune diseases related to high or low socioeconomic position are shown.

SES contributes to the observed difference in the prevalence of immunological disorders based on time and geographical distribution (Figure 8). As better public health measures have been taken since the industrial revolution, there has been a major decline in infectious diseases with a simultaneous increase of ADs. This has been cataloged as the hygiene hypothesis (196). According to this theory, social and environmental exposures have a direct impact on immune tolerance and response, supporting the association between SES and AD development.

In RA, different studies have reported a reduced risk as the educational and income level rise (197–199). Furthermore, a correlation was found between 11 years of education or less with a two-fold increase in poor prognosis (200). For instance, the lower the patient’s educational level, the greater the chance of mortality and the lower their functional capacity (201). In terms of self-assessment, health quality is usually low in patients with RA who are older, female, less educated, obese, unemployed, and less affluent than other groups (202, 203). Many RA patients tend to be unable to work due to their low functional capacity. Thus, they become dependent on the state for health services and social welfare support (204).

A recently published study was done on 1,209 RA patients to examine the association between SES and DMARDs treatment delays, disease activity, joint damage, and disability. Disease activity was determined by DAS28, joint damage was determined from hand radiographs by Sharp score, and physical disability was determined by MDHAQ. On average, patients with lower SES waited 8.5 ± 10.2 years after onset of RA symptoms to begin DMARD treatment compared to those in the middle and upper SES who waited 6.1 ± 7.9 years (*p* = 0.002) and 6.1 ± 8.6 years (*p* = 0.009), respectively. Moreover, patients with the greatest DMARD lag showed greater DAS28, Sharp, and MDHAQ scores (*p* < 0.001) (205).

In terms of mortality, a retrospective cohort study was done on the Taiwanese population to evaluate the association of individual SES/neighborhood SES, and mortality rates in 23,900 RA patients from 2004 to 2008. Analysis revealed that 5-year mortality rates were worse among RA patients with a low individual SES compared to those with a high SES (*p* < 0.001). Additionally, patients with low SES in disadvantaged neighborhoods had the highest risk of mortality (HR: 1.64, 95% CI: 1.26–2.13, *p* < 0.001) (206).

For SLE, there is a tendency to earlier onset and worse outcomes in people of Hispanic, Asian, or African ancestry compared to Caucasians. This may be partly related to overall poor SES, including less structured families, fewer years of formal education, occupational status, household income, higher poverty, and inadequate health insurance (199). The costs of SLE can be different depending on the health care system. The impact of indirect costs is influenced by poor physical and mental health, low social support, low educational level, unemployment, and high disease activity (199, 207). Moreover, patients with elevated mortality and incidence of end-stage renal disease reported greater levels of poverty (199).

A cross-sectional study done on Mexican SLE patients evaluated the relationship between SES and organ damage. Lupus activity and organ damage were measured using the SLEDAI and Systemic Lupus International Collaborating Clinics/American College of Rheumatology (SLICC/ACR) scale. The analysis included 143 SLE female patients, of which 42.7% presented organ damage. Patients with organ damage had lower monthly household incomes and were more frequently unemployed. Low monthly income was not associated with lupus activity or self-reported altered health status (208).

The relationship between low SES and cardiovascular risk factors in SLE has also been evaluated. A longitudinal cohort study included 1,752 SLE patients. Regression analyses stratified by ethnicity and low income were strongly associated with most traditional cardiovascular risk factors in Caucasians, but only with smoking and diabetes in African Americans. In Caucasians, low income increased the risk of both myocardial infarction (OR: 3.24, 95% CI: 1.41–7.45, *p* = 0.006) and stroke (OR: 2.85, 95% CI: 1.56–5.21, *p* = 0.001); in African Americans, these relationships were not seen. Moreover, low education was associated with smoking in both ethnic groups (209).

For MS, African Americans have been reported to start at a younger age, in comparison to Caucasians or Hispanics. Access to neurology consultation and rehabilitation services was determined by health insurance, poverty, and living in rural areas. Additionally, important improvements in quality of life related to educational level and employment status in MS patients were identified (210, 211).

In conclusion, SES has a major impact on health as it affects biological functions. In general, low SES has been associated with worse outcomes and prognosis in ADs. The biological relevance of SES relies on ancestry and environmental exposure (199).

### Sex Hormones, Gender, and Autoimmunity

Differences in AD manifestations by gender involve immunomodulation by sex hormones, non-hormonal factors encoded by genes on the X and Y chromosomes, and immunological phenomena unique to pregnancy (212). Over 75% of patients with ADs are estimated to be women, and hormones are important in regulating the onset, severity, and progression of the disease (213). In fact, AD is considered the fourth leading cause of disability for women (214).

Many studies have postulated that oral contraceptive (OC) exposure exacerbates SLE activity, but the evidence has been controversial. Two randomized controlled trials (RCTs) addressed this issue. The first RCT was single-blinded, and included 162 women with SLE who were randomly assigned to a combined OC (30 μg ethinyl estradiol), a non-eluting intrauterine device, or a progestin-only pill for 12 months. There was no statistical difference in the flare rate between the groups (215). The second RCT was double-blinded, and evaluated a combined OC (35 μg ethinyl estradiol) versus placebo for 12 months. It included 183 women with SLE with inactive disease, and analysis showed no statistical difference in flare rate (216).

**Figure 9:**
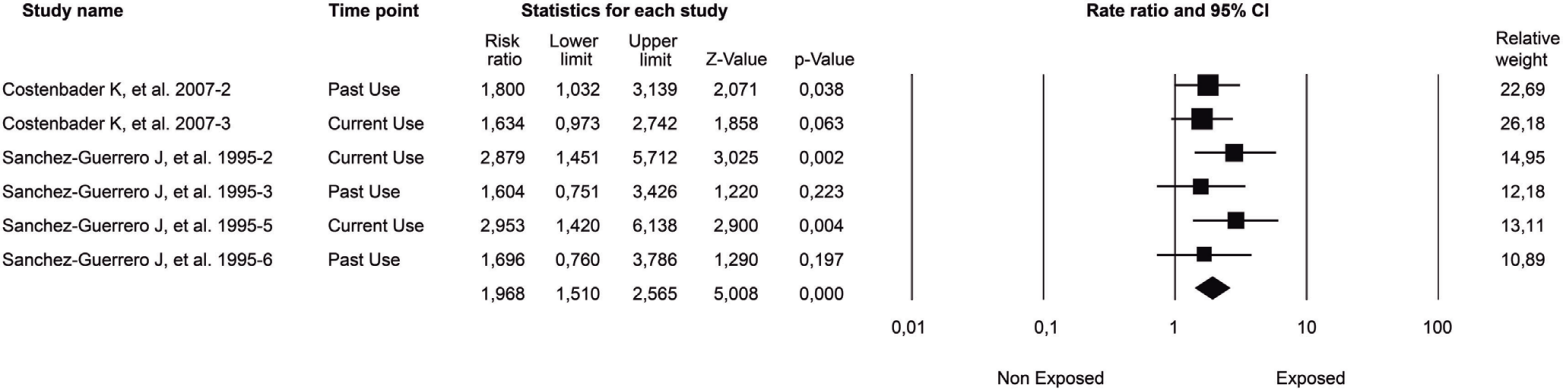
**Association between hormonal replacement therapy (HRT) exposure and risk of developing SLE**. Forest plot of studies meta-analyzed. Final common effect size based on a random model. CI: confidence interval; SLE: systemic lupus erythematosus; HRT: hormonal replacement therapy. Adapted with authors’ permission from Ref. (217).

A systematic review and meta-analysis evaluating the association between exposure to exogenous sex hormones (i.e., estrogens) and SLE (217), showed a significant association between hormonal replacement therapy (HRT) exposure and increased risk of SLE (RR: 1.96, 95% CI: 1.51–2.56, *p* < 0.001) (Figure 9). There was no association between HRT exposure and specific outcomes of disease. Six meta-analyses were run evaluating different outcomes: death, all flares, major flares, thrombosis, and coronary disease. None were significant (217). Moreover, analyses seeking an association between OC exposure and different SLE outcomes were not significant with the exception of a marginal result in a meta-analysis, including the SLE outcome for patients with “ever-use” status and stratified by age. In conclusion, HRT exposure increases the risk of SLE in healthy women. Considering the selection bias of the studies included in the meta-analysis, which in general, excluded women with high disease, or antiphospholipid antibodies (aPL-abs), or history of thrombosis, generalization of results in the present study was limited and the recommendations for using these on women with known SLE must be followed cautiously (217).

Another meta-analysis found no statistically significant association between OC exposure and RA risk (RR: 0.88, 95% CI: 0.75–1.03) (218).

Like SLE, SS and AITD are also highly prevalent among women (219). Several studies have recognized gender-associated characteristics of disease and severity. For instance, women with SS have more anti-Ro antibodies and Raynaud’s phenomenon than men (220). Additionally, men with SS are at higher risk for lymphoma and neurological involvement than women. Accordingly, male gender is considered a risk factor for lymphoma in patients with SS (213, 221, 222). Additionally, AITD represents the main cause of hypothyroidism during pregnancy ranging between 5 and 20% in prevalence with an average of 7.8% (223).

Larger trials are required to assess the long-term effects of hormone therapy (e.g., contraception or postmenopausal) on the course of ADs and to identify patient characteristics associated with an increased risk of flares in hormone therapy exposure.

### PolyA in the Context of Autoimmune Ecology

As previously mentioned, the fact that ADs share several clinical signs and symptoms (i.e., subphenotypes), physiopathological mechanisms, and genetic factors has been called autoimmune tautology (12). The clinical evidence of autoimmune tautology highlights the co-occurrence of distinct ADs within an individual and becomes known as PolyA. Although there are several studies evaluating AE through the various ADs, there are few clinical studies of specific shared environmental factors favoring PolyA (224). Some examples of specific associated factors are shown below.

#### Vitamin D Levels and PolyA

T1DM and MS, two organ-specific ADs, frequently occur together (i.e., PolyA) in the Sardinian population (225). Patients with MS in Sardinia have a sixfold higher risk of T1DM than healthy controls. Although the frequency of the risk HLA group (HLA-DRB1*03) associated with T1DM and MS is highly increased in Sardinia, this genetic group does not seem to account for all T1DM–MS PolyA. Rather, the environment may be the most relevant element determining this risk, perhaps in a setting where susceptibility haplotypes do not decrease the chance of co-occurrence of the two (226). Vitamin D is a shared environmental factor evaluated for these conditions. There is evidence showing a preventive role of vitamin D in MS and T1DM. A latitudinal gradient characterizes both diseases. T1DM and MS become increasingly common as distance from the equator increases and implicates common factors, such as Vitamin D deficiency (226). Although this environmental factor is shared, it has not been systematically evaluated in individuals who have this PolyA.

Recently, a study in celiac disease (CD) was done on 145 patients searching for PolyA prevalence and related risk factors. The study participants were divided into two groups. Group 1 was the CD-alone group consisting of patients without any other ADs, while group 2 was the PolyA group consisting of patients with one or more accompanying ADs. When the groups were compared in terms of demographic features and laboratory data, vitamin D deficiency was the environmental factor associated with PolyA along with the following risk factors: female gender, family history for ADs, antigliadin IgG positivity, antinuclear antibody positivity ≥1/80 titer, and any musculoskeletal disease (227).

A retrospective cross-sectional study enrolled 133 women with recurrent pregnancy loss (RPL) for a 2-year period together with laboratory experiments (228). Sixty-three of them (47.4%) had low vitamin D levels (<30 ng/ml). The prevalence of aPL-abs (anticardiolipin IgG and IgM) was significantly higher in the low vitamin D group (VDlow) (39.7%) than in the normal group (VDnl) (22.9%) (OR: 0.22, 95% CI: 1–4.7, *p* = 0.05). Furthermore, anti-ssDNA (19.0 versus 5.7%, OR: 3.76, 95% CI: 1.1–12.4, *p* = 0.05) and anti-thyroperoxidase antibody (33.3 versus 15.7%; OR: 2.68, 95% CI: 1.2–6.1, *p* = 0.05) were significantly higher in VDlow than in VDnl. Although authors did not study the clinical confirmation of AITD, antiphospholipid syndrome (APS), and SLE through validated clinical criteria in the whole group, a serologic PolyA of AITD and SLE is displayed in a highly APS-potential group of patients (RPL with aPL-abs positivity). Since autoantibodies are predictors of disease, it is important to vigilantly following the clinical course of patients with RPL and VDlow for prediction of PolyA (APS plus AITD and SLE).

For APS and SLE PolyA, a relationship between vitamin D levels and the co-ocurrence of these diseases has also been evaluated. Serum vitamin D levels were measured in 179 European APS patients and 141 controls (229). Of these, 113 APS patients were diagnosed with primary APS (pAPS) and 66 with the previously so-called APS secondary to SLE (i.e., PolyA). The mean levels differed between patients with APS alone (18 ± 9 ng/ml) and PolyA with SLE who disclosed lower levels of vitamin D [14 ± 8 ng/ml (*p* = 0.004)]. However, upon individual analysis, both levels were significantly lower than that for the controls.

Schoindre et al. (230), evaluated serum 25(OH) Vitamin D levels in 170 patients with SLE who were prospectively followed up for 6 months. In a multivariate analysis contrasting with the previously mentioned results, absence of defined APS (*p* = 0.002) was one of the predictive factors of lower 25(OH)D levels, which showed an opposite relationship between low Vitamin D levels and PolyA with APS. These contrasting results should be analyzed carefully because there are few studies evaluating the influence of vitamin D levels in APS–SLE PolyA.

Similarly, Schneider et al. (231) evaluated 25(OH)D levels and cytokine profiles in 172 patients with SLE. There were 11 patients (6.4%) having PolyA with SS and the same number with APS. Although the main focus of the study was not the 25(OH)D levels in those PolyA patients, no associations were found between the presence of the previously so-called secondary APS nor secondary SS and vitamin D concentration.

All of the above results are linked to the fact that Vitamin D interacts with the immune system. It takes part in the regulation and differentiation of immune system cells directly and indirectly. Current data link vitamin D deficiency to many ADs, including T1DM, MS, inflammatory bowel disease, SLE, and RA. It is essential for part in the genetic regulation of cytokine production, VDR expression and affects biological processes by which these cells interact. Overall, vitamin D confers an immunosuppressive effect. Vitamin D has been shown to be clinically beneficial for animal models, and initial observations indicate that vitamin D supplementation may help prevent MS and T1DM (232). Further research is expected regarding the interaction between Vitamin D levels and PolyA.

#### Cigarette Smoking and PolyA

There are few or no studies designed to evaluate the relationship between cigarette smoking and PolyA. Nevertheless, this association has been evaluated as a secondary end point in studies researching PolyA globally.

A recent publication (233) analyzed the association between smoking and aPL-abs in SLE patients and explored the relationship between smoking, aPL-abs, and vascular events (arterial and venous, VE). Out of a total of 367 SLE patients evaluated, 118 were aPL-abs positive at inclusion. In regression-adjusted models, regular smoking was associated with aCL IgG, aβ2GP1 IgG, LA, and triple aPL-abs positivity. Furthermore, former and current smokers were more likely to have a history of arterial events than never smokers.

A possible explanation for these results is that smoking may contribute to oxidative alterations, which, in genetically predisposed individuals, may give rise to an autoimmune response, epitope spreading, and induction of aPL-abs resulting in the SLE–APS PolyA and its association with cardiovascular events.

There are various studies showing the independent relationship between smoking, APS or aPL-abs, and the specific cardiovascular compromise subphenotype in SLE patients. Recently, Fernández-Nebro et al. (234), identified smoking (OR: 1.48, 95% CI: 1.06–2.07) and aPL-abs (OR: 1.57, 95% CI: 1.13–2.17) among other independent variables as risk factors for CV events through a multivariate analysis. The study involved 374 SLE patients (10.9%) from a total of 3,658 patients, who suffered at least one CV event. In addition, Ahmad et al. (235) evaluated the strength of association between traditional cardiovascular risk factors and carotid plaque development in 200 women with SLE and 100 controls. When combined in a final model, classic and SLE-related factors, they showed pack-years of smoking as well as serologic PolyA (aCL and/or any LAC) among other variables (any azathioprine exposure, age, and previous arterial events) as predictors of subclinical atherosclerosis. Likewise, in a multi-ethnic SLE cohort (LUMINA) of 570 patients (236), 51 developed at least 1 venous thrombotic event after SLE diagnosis. Through a cox proportional hazard model, an independent association with smoking and positivity for LAC was demonstrated among other variables related to venous thrombosis.

Additionally, an independent association between APS PolyA (OR: 4.71, 95% CI: 1.81–12.2, *p* < 0.001) and smoking/coffee interaction (OR: 1.82, 95% CI: 1.05–3.13, *p* = 0.03) with CVD was previously shown in a cohort of 310 patients with SLE through a logistic regression model (166).

**Figure 10:**
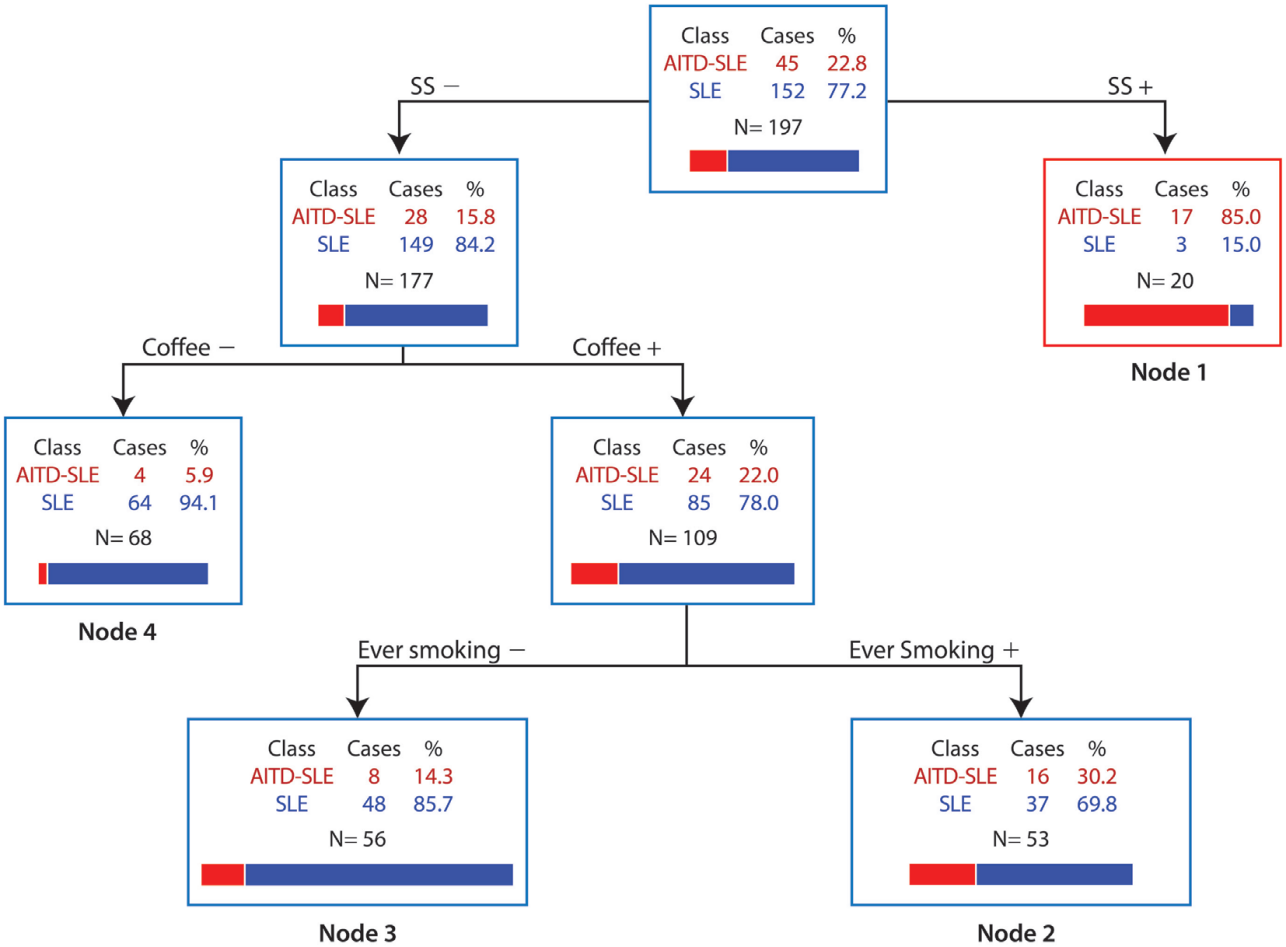
**Predictive factors associated with AITD PolyA in SLE**. Sjögren syndrome and ever smoking are the main factors associated with the development of AITD in SLE. AITD, autoimmune thyroid disease; PolyA, Polyautoimmunity; SLE, systemic lupus erythematosus; SS, Sjögren’s syndrome. Adapted from Ref. (237).

In a Colombian cohort of 376 patients with SLE (237), we evaluated the PolyA with autoimmune hypothyroidism and its related factors. Multivariate analysis and a classification and regression tree model (CART) were used to analyze data. After adjusting for gender and duration of disease, ever smoking (AOR: 6.93, 95% CI: 1.98–28.54, *p* = 0.004), and PolyA with SS were persistently associated with AITD. Ever-smoking exposure as environmental factor was confirmed as a main factor associated with AITD PolyA in SLE patients through the CART model (Figure 10).

The association between smoking exposure and PolyA has been seen not only in SLE but also in other ADs. For example, the coexistence of ADs (i.e., PolyA) in SS and related factors was evaluated earlier in a cross-sectional study involving 410 patients. Of these, 134 (32.6%) had PolyA. In the regression analysis, duration of disease, positive smoking status (AOR: 2.86, 95% CI: 1.18–6.94, *p* = 0.02), and a history of spontaneous abortions remained significantly associated with PolyA (238).

Lastly, 955 consecutive RA patients (1987 ACR criteria) and their nuclear families were included in a single-center cohort study (239). The history of 23 ADs, the factors related to PolyA, and familial autoimmunity were investigated. The mixed cluster methodology, based on multivariate descriptive methods, was used to summarize sets of related variables with strong associations and common clinical context. Then, for each set of related variables, new cluster variables (NCV) were derived for each patient. This was done on two sets of variables: the first were variables related to toxic substances consumed by patients from which we derived the toxic consumption profile as NCV; and the second was related to severity variables from which we derived the NCV of severity profile. The *X*^2^ and Fisher’s exact tests were done to establish differences between categorical variables (original and NCV) and PolyA. There were 130 (13.6%) patients who met the classification criteria for at least one other AD. Main factors influencing PolyA were familial autoimmunity, familial RA, and toxic consumption profile. For toxic consumption profile NCV, the proportion of PolyA in RA patients increases with more severe toxic exposure (no toxic consumption, followed by just coffee consumption exposure, then by coffee plus tobacco exposure, and finally, by coffee consumption plus hair dye).

**Figure 11:**
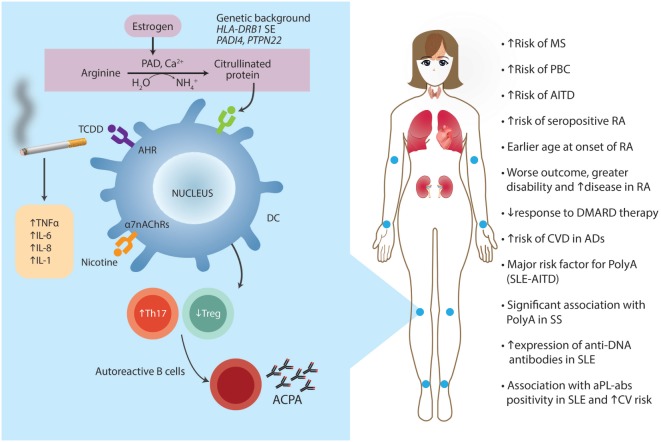
**Cigarrete smoking and autoimmunity**. In predisposed individuals carrying certain autoimmune alleles at *HLA-DRB1, PTPN22*, and *PADI4* genes, cigarette smoke triggers citrullination by activating PAD enzyme, a mechanism that is estrogen dependent. Citrullinated proteins act as auto-antigens and induce the formation of anticitrullinated protein autoantibodies (ACPAs) by autoreactive B cells. 2,3,7,8-tetrachlorodibenzo-p-dioxin (TCDD), released during cigarette smoking, acts as the best-known ligand for aromatic hydrocarbon receptor (AhR). AhR activation induces boosts T helper 17 (Th17) cells. Nicotine stimulates the α7-nicotine acetylcholine receptors (α7nAChRs), and increases TNFα, IL-6, IL-8, and IL-1 synthesis. The effect on T regulatory (Treg) function is controversial. All of these molecular pathways are responsible for the clinical manifestations listed on the right. PAD, peptidylarginine deiminase; SE, shared epitope; DC, dendritic cell; MS, multiple sclerosis; PBC, primary biliary cirrhosis; AITD, autoimmune thyroid disease; RA, rheumatoid arthritis; DMARD, disease-modifying antirheumatic drug; CVD, cardiovascular disease; ADs, autoimmune diseases; PolyA, polyautoimmunity; SLE, systemic lupus erythematosus; SS, Sjögren syndrome; aPL-abs, antiphospholipid antibodies.

A summary of the effects of cigarette smoking on the development of ADs is shown in Figure 11.

#### Socioeconomic Status, Infections, and PolyA

As mentioned previously, several factors, such as SES, may explain differences in the prevalence of immunological disorders based on time and geographical distribution. Research on the influence of SES in PolyA is scarce. The influence of SES on the course of ADs in admixed and heterogeneous socio-demographic populations was evaluated in a cross-sectional analytical study in which 1,096 consecutive patients with RA (*n* = 678), SLE (*n* = 258), and SS (*n* = 160) were included. Significant association between low SES and a stronger autoimmune response was observed (240). In addition, low SES influences the presence of arthritis, Raynaud’s phenomenon, and discoid lupus in SLE. As expected, an association between low SES and low educational level was observed in SLE and SS; while in RA, a low SES was also associated with housewives. The association between PolyA and low SES in SS patients (OR: 3.692, 95% CI: 1.49–9.18, *p* = 0.005) should be highlighted. These results could be related to the admixture of the evaluated population, environmental factors, or the quality of health insurance and deserve further research. The results of this study contrast with those of others. For instance, PolyA between RA and AITD plus associated factors was evaluated in a cohort of 800 RA patients (241). The prevalence of AITD in RA patients was 9.8%. A lower AITD frequency was found in the lowest educational level compared to the highest one. Therefore, a low educational level (a surrogate of low SES) was positively associated with AITD–RA PolyA (OR: 0.16, 95% CI: 0.03–0.88, *p* = 0.036). These results should be analyzed in the context of the mentioned “hygiene hypothesis” or a high infectious agent exposure (exposure is interpreted as a poor hygiene problem). Epidemiological studies indicate a direct link between the decreasing infectious burden and the rising incidence of immunological disorders (196). Also, social and environmental exposures directly impact immune tolerance and response. Furthermore, several epidemiological studies have investigated the protective effect of infectious agents in allergy and ADs.

Although the relationship between infectious disease and autoimmunity was not intended to be part of this review, some examples regarding the association between infectious disease and PolyA are shown below. EBV and CMV are notorious as they are consistently associated with multiple ADs. A cohort of 1,595 serum samples from 23 different AD groups was screened for evidence of prior infection with EBV and CMV (242). When compared to healthy controls, a significant increase in titers of EBV Viral Capsid Antigen IgG was observed in sera of patients with SLE–APS PolyA as well as in both diseases separately and in patients with other, single ADs (e.g., RA, MS, etc.). Moreover, samples from 82 consecutive SS patients, and 139 healthy controls were analyzed for infectious serology and autoantibodies as well as for relevant genetic mutations (TAP genes) and cytokine levels (243). Vasculitis PolyA among SS patients was significantly related to the presence of *Saccharomyces cerevisiae* antibody IgG (OR: 6.5, 95% CI: 1.14–36.78, *p* = 0.05). No other infectious autoantibodies were related to PolyA.

In contrast to this, when evaluating sera from 260 individuals (120 SLE patients, 140 geographically matched controls) for titers of infectious antibodies (five EBV-Abs), differences in titers from SLE-associated APS were not detected (244).

The results presented here agree with clinical evidence for all of the current theories on how infections could cause AD. Recent evidence suggests that microbe-activating specific innate immune responses are critical while antigenic cross-reactivity may perpetuate immune responses leading to chronic autoinflammatory disease (245). The data regarding cross-talk between PolyA as an expression of specific activated immunological pathways and infectious agents suggest that infections may play both a causative role and a protective role in the pathogenesis of ADs.

### Organic Solvents and Autoimmunity

Solvents are liquids that dissolve a solid, liquid, or gas and are broadly classified into two categories: organic and inorganic. OSs are compounds with carbon-containing molecules. They may be broken down further into aliphatic-chain compounds, such as n-hexane, and aromatic compounds with a six-carbon ring, such as benzene or xylene. Common uses of OSs are dry cleaning (e.g., tetrachloroethylene), paint thinner (e.g., toluene, turpentine), nail polish removers, and glue solvents (acetone, methyl acetate, ethyl acetate), spot removers (e.g., hexane, petrol ether), detergents (citrus turpenes), perfumes (ethanol), nail polish, and chemical synthesis, etc. (246). OSs are capable of altering cellular proliferation, apoptosis, and tissue-specific function. Both the amount and duration of OS exposure are essential in pathology causality. Chronic exposure to OSs might lead to deposits in an organ and consequently to immune infiltration similar to what is observed in ADs. The self-proteins that are modified by OSs may become immunogenic, recognized as foreign and, thus, initiate an inflammatory response and tissue injury (246).

Research in this area began in the 1950s after case reports of patients who developed a scleroderma-like syndrome after exposure to vinyl chloride, epoxy resins, trichloroethylene, perchloroethylene, or mixed solvents (11). The evidence of an association between exposure to OSs and developing AD was recently analyzed through a systematic literature review and meta-analysis. Even though the individual meta-analyses (i.e., each AD considered separately) showed significant association for MS, primary systemic vasculitis, and SSc, the direction and significance of this association did not change when all ADs, considered as a single trait, were analyzed (OR: 1.54, 95% CI: 1.25–1.92, *p* = 0.001). To our knowledge, this is the first evaluation of all ADs as a group with respect to OSs exposure through a meta-analysis. Therefore, the fact that ADs might share several common mechanisms (i.e., the autoimmune tautology) is reinforced (246).

Nevertheless, a recent review of systematic reviews and meta-analyses evaluating environmental risk factors and MS did not show a significant association (247). Likewise, the occurrence of MS among subjects exposed to anesthetic agents was compared to that of those who have never been exposed. This was based on previous evidence that exposure to anesthetic agents, some of which are chemically related to organic solvents, may affect the risk of MS. Therefore, based on two population-based, case-control studies, one with incident cases (1,798 cases, 3,907 controls) and one with prevalent cases (5,216 cases, 4,701 controls), no association was found between occupational exposure to anesthetic agents and risk of MS (248). By contrast, other studies evaluating the relationship between occupational exposure and risk of MS have shown positive associations. In a matched case-control study of 276 first clinical diagnosis of central nervous system demyelination cases and 538 controls done in Australia (2003–2006), Valery et al. examined the association between occupational exposure and risk of central nervous system demyelination. Among women, there was an increase in central nervous system demyelination risk associated with 10 or more years of exposure to livestock (AOR: 2.78, 95% CI: 1.22–6.33) or 6 or more years of farming (AOR: 2.00, 95% CI: 1.23–3.25; adjusted for number of children) (249). Horwitz et al. estimated the occupational risks in relation to MS in an open insurance cohort (all payouts for critical illness insurance from 2002 to 2011 were continuously registered). The MS incidence showed signs of occupation having an overall effect on the risk of MS. The high frequency found within the agricultural segment was attributed to dairy operators, whose incidence of MS was 2.0 times higher than the rest of the study’s population (95% CI: 1.2–3.0) (250). Furthermore, Magyari et al., studied whether occupation or physical or social environment influenced the risk of MS differently in women than in men in a cohort consisting of 1,403 patients (939 women, 464 men), identified through Danish MS registry (2000 and 2004), and up to 25 matched controls (251). They found a slight albeit statistically significant excess for six female MS patients who had been employed in agriculture: OR: 3.52; 95% CI: 1.38–9.00, *p* = 0.008 (0.046 when corrected for multiple significance) although the number of cases was small.

Concerning SSc, recent publications have shown an association between OSs exposure and SSc. Marie et al. (252) evaluated the relationship between SSc and occupational exposure between 2005 and 2008 in 100 patients with a definite diagnosis of SSc and 3 matched controls for each patient. Higher ORs for SSc were found for white spirit (*p* = 0.0001), aromatic solvents (*p* = 0.0002), chlorinated solvents (*p* = 0.014), trichlorethylene (*p* = 0.044), ketones (*p* = 0.002), and welding fumes (*p* = 0.021). In addition, in a prospective study involving 142 SSc patients, Marie et al. (253) showed that patients exposed to solvents more frequently developed diffuse cutaneous SSc (*p* = 0.001), digital ulcers (*p* = 0.01), interstitial lung disease (*p* = 0.02), myocardial dysfunction (*p* = 0.04), and cancer (*p* = 0.003). Moreover, these patients were more frequently anti-Scl 70 positive and anticentromere negative. Furthermore, based on those results, some researchers have recently suggested that the association between SSc and occupational exposure be legally recognized as an occupational disorder (254).

Lastly, Li et al. (255) investigated possible associations between occupation and hospitalization for SLE in a nationwide database (Swedish Census to the Hospital Discharge Register 1970–2008). A total of 8,921 male and 42,290 female hospitalizations for SLE were analyzed. Higher risks [standardized incidence ratios (SIR)] among men with the same occupation (SIR > 2.0) were present among workers exposed to OSs in two consecutive censuses: shoe and leather workers (6.93), plumbers (2.21), chimney sweeps (4.54), and military personnel (3.01), etc.

To summarize, the recent expert panel (10) was confident regarding evidence supporting an association between solvent exposure and developing SSc. They agreed that general solvent exposure may also contribute to MS, but more research is needed using improved exposure assessment methods.

In conclusion, an association between OSs exposure and ADs is observed. OS exposure has not yet been sufficiently studied, and to clarify its role in ADs pathogenesis, its relationship with genetics needs to be studied, whether with respect to protection or susceptibility to each AD and the effects on the autoimmune process.

### Vaccines

Vaccines represent the most successful and sustainable tactic to prevent and counteract infection. A vaccine improves immunity to a particular disease upon administration by inducing specific protective and efficient immune responses in all of the receiving population. The main known factors influencing the observed heterogeneity for immune responses induced by vaccines are gender, age, ethnicity, co-morbidity, immune system, and genetic background (256).

Autoimmunity is a concern for many vaccines, although AD presentation among immunized individuals is rarely observed. Nevertheless, and as mentioned, ASIA entails autoimmune conditions appearing after the exposure to an external stimuli of an adjuvant, including vaccines (257). In spite of some controversy about the diagnosis and classification criteria of this syndrome (258), we have observed and discussed patients who developed autoimmune conditions after quadrivalent human papillomavirus vaccination (259). However, because of relatively low baseline incidence of many autoimmune conditions, large post-marketing and adequately powered studies are required to evaluate any increased risk of ADs after vaccination. In fact, in most of the clinical trials evaluating vaccines, a systematic screening for ADs is not performed (i.e., testing for autoantibodies and evaluation of familial autoimmunity).

The incidence of narcolepsy, a sleep disorder characterized by loss of hypothalamic hypocretin (orexin) neurons, was increased after the pandemic AS03 adjuvanted H1N1 vaccination in Swedish and Finish but not in Italians (260). The clue for the observed side effects could be related to one type of vaccine, since not all the vaccines against H1N1 were associated with such complication. In fact, the vaccine inducing narcolepsy was suspected to trigger antibodies that can also bind hypocretin receptor 2 (261).

There is no a single mechanism explaining how a vaccine may induce AD. Theoretically, vaccines could trigger autoimmunity by means of cytokine production, anti-idiotypic network, expression of human histocompatibility leukocyte antigens, modification of surface antigens and induction of novel antigens, molecular mimicry, bystander activation, epitope spreading, and polyclonal activation of B cells (262).

There are several case reports of ADs following vaccines (45); however, due to the limited number of cases, the different classifications of symptoms, the long latency period of the diseases, and bias in data interpretation, every attempt for an epidemiological study has so far failed to deliver a connection. Despite this, efforts to unveil the connection between the triggering of the immune system by vaccines and the development of ADs should be undertaken (257).

On the other hand, in patients with AD receiving immunosuppressive therapy, vaccinations are often not offered or provided for a variety of reasons, including the fear of complications or vaccine-related illnesses, a concern for disease flare or reactivation, a perceived lack of effectiveness, or a misunderstanding of current vaccine guidelines (263). Patients with ADs often show decreased immune responsiveness, which in turn would make them vulnerable to infection given their underlying disease and frequent use of immunosuppressive drugs. A recent review highlights that all recommended vaccines may be given to patients before the start of immunosuppressive therapy (264, 265). All inactivated-killed vaccines should be provided to patients with AD, as they provide significant morbidity and mortality benefits. Inactivated-killed vaccines have not been shown to be associated with disease flares in large studies adequately powered to determine this effect. Live vaccines should be used mainly before the start of immunosuppressive therapy and used with caution on patients on active immunosuppressive therapy based on national guideline recommendations (264).

### Conclusions and Perspectives: Personalized Medicine and Risk Prediction

Personalized (or precision) medicine, taking account of human individuality, has shown great promise to transform medical care. Since most of genetic factors associated with ADs confer a modest risk of ADs (266) and the immune system is very much shaped by the environment (13), inclusion of the AE in disease etiology and health will improve the way personalized medicine is currently conceived and applied (267).

Better understanding of the complex gene–environment interactions involved in the development of ADs together with the study of epigenetics (which was beyond the scope of this review) will provide insight into personalized interventions for these common and sometimes devastating diseases. Algorithms, constructed based on data gathered at the population level, can shed light on how the parts work together and the causal relationships between them, allowing strategies for prediction and prevention of ADs.

## Author Contributions

Study conception and design: J-MA, AR-V. Analysis and interpretation of data: J-MA, CR-S, MA, NM-G, AR-V. Drafting of manuscript: J-MA, CR-S, MA, AR-V. Critical revision: J-MA, CR-S, MA, NM-G, AR-V.

## Conflict of Interest Statement

This research was conducted in the absence of any commercial or financial relationships that could be construed as a potential conflict of interest.

## Abbreviations

AA, adjuvant-induced arthritis; aa, amino acids; ACPAs, anticitrullinated protein autoantibody; ADs, autoimmune diseases; AE, autoimmune ecology; AhR, aromatic hydrocarbon receptor; AITD, autoimmune thyroid disease; ANCA, anti-neutrophil cytoplasmic autoantibody; AOR, adjusted odds ratio; aPL-abs, antiphospholipid antibodies; APS, antiphospholipid syndrome; ASIA, autoimmune/inflammatory syndrome induced by adjuvants; BAFF, B-cell activating factor; CD, celiac disease; CFA, complete Freund’s adjuvant; CIA, collagen-induced arthritis; CMV, cytomegalovirus; CrD, Crohn’s disease; CREB, cAMP response element-binding protein; CVD, cardiovascular disease; DAMPs, damage-associated molecular patterns; DAS 28, disease activity score-28; DCs, dendritic cells; DEPAP, 1,2-di-oleyl ester; DMARDs, disease-modifying antirheumatic drugs; dmC, deoxymethylcytosine; DNMT1, DNA methyltransferase 1; dsDNA, double-strain DNA; EAE, experimental autoimmune encephalomyelitis; EBV, Epstein–Barr virus; EPW, expert panel workshop; ERK, extracellular signal-regulated kinase; EULAR, European league against rheumatism; FICZ, 6-formylindolo (3.2-b) carbazole; GC, germinal center; GD, Graves’ disease; GO, Graves opthalmopathy; GSE, gluten-sensitive enteropathy; GST, glutathione S-transferase; HMGB1, high mobility group box 1; hnRNP, heterogeneous nuclear ribonucleoprotein; HR, hazard ratio; HRT, hormonal replacement therapy; HT, Hashimoto’s thyroiditis; IFN, interferon; Igg, immunoglobulin; IRAK1, interleukin 1 receptor associated kinase 1; IRF, interferon regulatory factor; LN, lupus nephritis; LPS, lipopolysaccharide; MAPKs, mitogen-activated protein kinases; MD, mean difference; MDHAQ, multi-dimensional health assessment questionnaire; MHC, major histocompatibility complexes; MS, multiple sclerosis; MTX, methotrexate; MYD88, myeloid differentiation primary response protein 88; NCV, new cluster variables; NF-κB, nuclear factor-κB; NLRs, nod-like receptors; OC, oral contraceptive; OR, odds ratio; OSs, organic solvents; PAD, peptidylarginine deiminase; PAMPs, pathogen-associated molecular patterns; pAPS, primary antiphospholipid syndrome; PBC, primary biliary cirrhosis; PBMC, peripheral blood mononuclear cells; PolyA, polyautoimmunity; PRR, pattern recognition receptors; PSC, primary sclerosing cholangitis; PTMs, post-translational modifications; RA, rheumatoid arthritis; RCTs, randomized controlled trials; RF, rheumatoid factor; RPL, recurrent pregnancy loss; RR, relative risk; SE, shared epitope; SES, socioeconomic status; SHM, somatic hypermutation; SIR, standardized incidence ratios; SLE, systemic lupus erythematosus; SLEDAI, systemic lupus erythematosus disease activity index; SLICC/ACR, Systemic Lupus International Collaborating Clinics/American College of Rheumatology; SMD, standardized mean difference; snRNA, small nuclear RNA; snRNP, small nuclear ribonucleoprotein; SPD, secondary progressive disease; SS, Sjögren’s syndrome; SSc, systemic sclerosis; T1DM, type 1 diabetes mellitus; TAK1, TGFβ-activated kinase 1; TCDD, 2,3,7,8-tetrachlorodibenzo-p-dioxin; Th17, T helper 17 cells; TIRAP, TIR domain-containing adaptor protein; TLR, toll-like receptor; TNF, tumor necrosis factor; TRAF6, TNF receptor associated factor 6; TRAM, TRIF-related adaptor molecule; Treg, T regulatory cells; Tresp, T-responder cells; TRIF, TIR domain-containing adaptor protein inducing IFNβ; UC, ulcerative colitis; UV, ultraviolet.
